# A systematic review and meta-analysis of the use of human albumin solution for treating the complications of liver cirrhosis

**DOI:** 10.1097/HC9.0000000000000875

**Published:** 2026-01-16

**Authors:** Reem M. AlSowaiegh, Khalifa A. Yusuf, Nick Freemantle, Alastair O’Brien

**Affiliations:** 1 Comprehensive Clinical Trials Unit, University College London, London, UK; 2 Department of Internal Medicine, Bahrain Defence Force Hospital, Kingdom of Bahrain

**Keywords:** humans, liver cirrhosis, renal insufficiency, serum albumin, randomized controlled trials

## Abstract

**Background:**

Decompensated liver cirrhosis is characterized by serious complications and high mortality. Intravenous human albumin solution (HAS) has been used for decades to treat these complications, and this systematic review aims to evaluate the clinical effectiveness and safety of HAS and compare it with alternatives.

**Methods:**

We searched MEDLINE (Ovid), Embase (Ovid), and CENTRAL (Cochrane Central Register of Controlled Trials) for Randomized Controlled Trials (RCTs) of HAS to treat liver cirrhosis. RCTs evaluating the use of HAS in cirrhotic adults were eligible. The clinical effect of protocol-guided HAS compared with alternatives on mortality was our principal outcome. Secondary outcomes included renal dysfunction, hepatic encephalopathy, gastrointestinal bleeding, portal hypertensive bleeding, recurrence of ascites, bacterial infections, paracentesis-induced circulatory dysfunction, multiorgan failure, and acute-on-chronic liver failure. Random effect odds ratios (ORs) and their confidence intervals (CIs) were reported.

**Results:**

Sixty-eight RCTs were included, with 4 evaluated as low risk of bias. Fifty-six RCTs reported mortality, with a statistically significant reduction in the overall odds of death compared with alternatives. The overall pooled random OR was 0.769 (95% CI 0.652–0.908), *p*=0.0019. Subgroup analyses grouping trials by size showed that only those with <100 participants demonstrated a significant reduction in overall odds of death (31.3%). HAS reduced the overall OR of renal dysfunction and recurrent ascites.

**Conclusions:**

HAS has shown a reduction in mortality and several complications. However, these benefits were primarily observed in small, low-quality trials, suggesting the need for large, well-designed RCTs before definitive recommendations can be made.

## Graphical abstract

**Figure:**
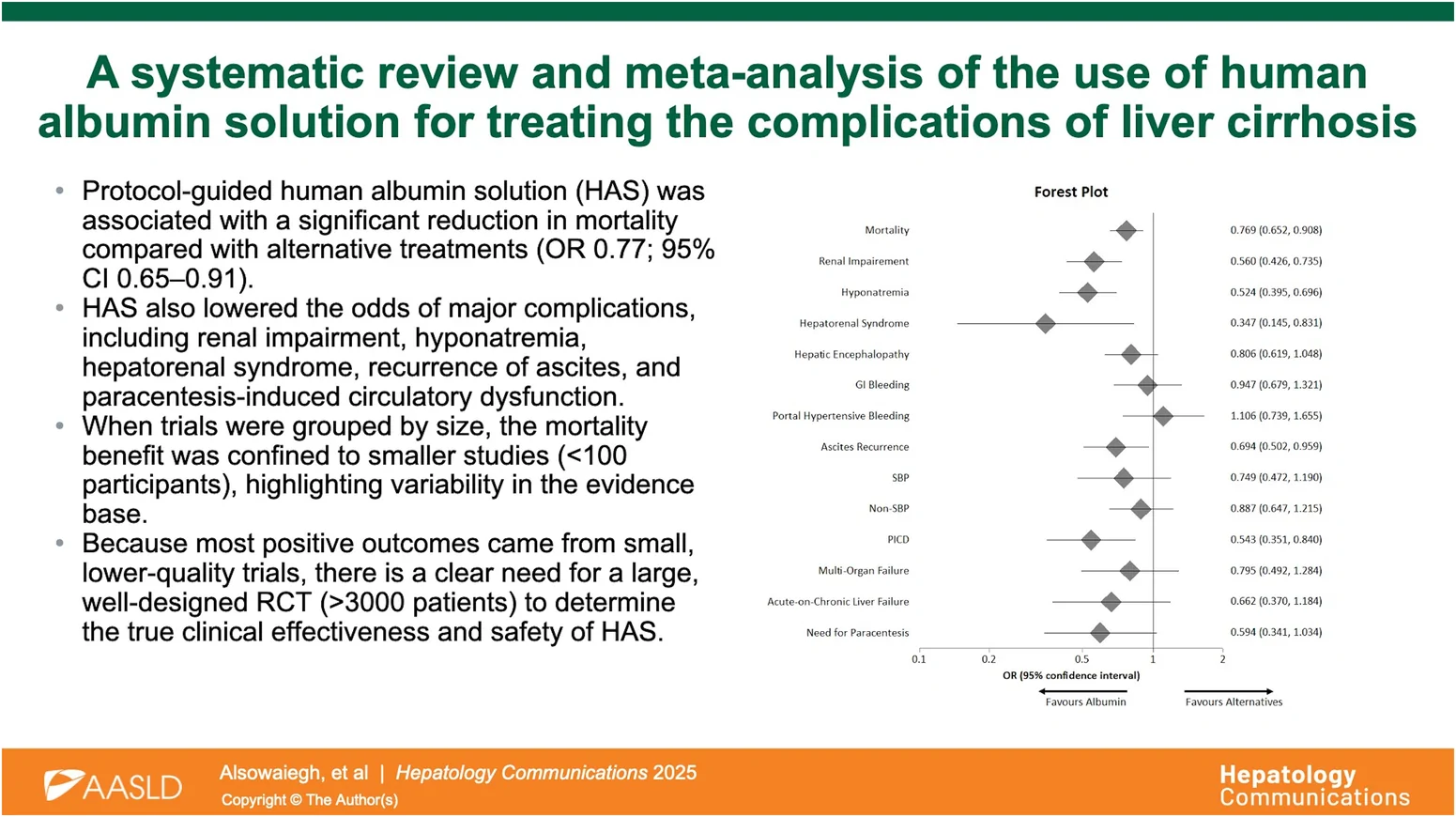

## INTRODUCTION

Over 45 million people are diagnosed with liver cirrhosis globally, leading to 2 million deaths annually.^1^^,^^2^ It is the ninth commonest cause of death in the United States and the second commonest cause of death in those aged 35–50 in England and Wales.^3^^,^^4^

Liver cirrhosis causes peripheral arterial dilatation leading to circulatory dysfunction and causes increased systemic inflammation, which leads to complications such as renal impairment, hyponatremia, hepatorenal syndrome (HRS), hepatic encephalopathy (HE), gastrointestinal bleeding, ascites recurrence, infections, paracentesis-induced circulatory dysfunction (PICD), and multiorgan failure, which are associated with high mortality.^5^^–^^7^ Intravenous human albumin solution (HAS) is a plasma expander that can restore normovolaemia, has potential anti-inflammatory properties, and is widely used to treat these complications.^2^^,^^6^

Several systematic reviews have investigated the use of HAS to manage liver cirrhosis-related complications.^5^^–^^11^ Recent meta-analyses by Bai et al.^7^ and Leache et al.^6^ reported that HAS can decrease mortality, renal impairment, and ascites of liver cirrhotic patients. In contrast, Shrestha et al.^5^ and Xu et al.^11^ showed that short-term and long-term HAS administration did not reduce the mortality of patients with decompensated liver cirrhosis. In view of these differing findings, we aimed to update, re-evaluate, and rationalize the clinical effectiveness and safety of HAS and compare it with alternative therapies for the treatment of the complications of liver cirrhosis to inform future clinical practice and guideline development.

## METHODS

### Registration

This work was registered in the International Prospective Register of Systematic Reviews PROSPERO 2023 (registration number CRD42023425220) and performed following the Preferred Reporting Items for Systematic Reviews and Meta-Analyses (PRISMA) 2020 checklist (Supplemental Materials, http://links.lww.com/HC9/C224). Ethics approval was not required. This systematic review was exempted from Institutional Review Board approval as the study did no involve humans.

### Selection of studies (search strategy)/literature search

We searched MEDLINE (Ovid), Embase (Ovid), and CENTRAL (Cochrane Central Register of Controlled Trials) to locate randomized controlled trials (RCTs) of HAS to treat adults with liver cirrhosis. The databases were searched from inception to April 2, 2025. We also used PubMed for preliminary scoping searches.

We searched 3 concepts/domains: liver cirrhosis, HAS, and RCTs. We identified controlled vocabulary terms, synonyms, indexed terms, and free-text terms for each concept and Subject Headings (MeSH and EMTREE), where applicable. We linked the MEDLINE search to the validated Cochrane Handbook Highly Sensitive Search Strategy filter (HSSS) for identifying RCTs, which we then adapted for the Embase search. We imposed no limits on date or language on the search. The full formal search strategy is available in the Supplemental Materials, http://links.lww.com/HC9/C224.

### Selection criteria

#### Inclusion criteria

1. RCTs
2. All liver decompensated cirrhosis complications
3. HAS is extracted from donated blood (human albumin solution: HAS)
4. Trials including additional interventions to HAS
5. All languages/no language restrictions (to exclude publication bias)
6. Adult population
7. Full-text articles and abstracts with enough detail for outcome data extraction.

#### Exclusion criteria

1. Non-randomized studies (eg, observational studies/retrospective studies/case-controlled studies/cross-sectional studies/case reports)
2. Interventional studies with these titles (these are explicitly titled and not aetiologies of liver cirrhosis complications):
  - Cancer/tumor/malignancy (eg, hepatobiliary cancers, hepatocellular carcinoma, cholangiocarcinoma)
  - Hepatitis (B and C)
  - Antiviral therapy [eg, ribavirin, interferon alpha, direct-acting antiviral (DAA)] and UDCA (ursodeoxycholic acid)
  - Primary biliary cirrhosis/primary biliary cholangitis/primary sclerosing cholangitis
  - NASH (non-alcoholic steatohepatitis)/non-alcoholic fatty liver disease
  - Herbal treatments/interventions (eg, Chinese medicine/homeopathies)
  - Liver fibrosis
  - Transplant/transplantation
  - Gene therapy
3. Non-human HAS
4. Pre-clinical trials
5. Children/pediatrics/pediatrics
6. Gene polymorphism studies
7. Tyrosine kinase inhibitors
8. Scoring systems (cancer scoring systems/propensity score matching scoring systems)
9. HIV
10. Duplicate studies
11. Guidelines
12. Systematic reviews or meta-analyses.

The RCTs in which inpatients or outpatients with advanced liver cirrhosis received intravenous HAS in the treatment arm were selected and compared with alternatives.

### Endpoints

Principal outcome: Mortality

Secondary outcomes:

- Binary/dichotomous outcomes
  1. Renal dysfunction
    - Renal impairment
    - Hyponatremia
    - HRS
    - PICD
  2. HE
  3. Gastrointestinal bleeding
    - Portal hypertensive bleeding (including variceal bleeding)
  4. Recurrence of ascites
  5. Development of bacterial Infection [including spontaneous bacterial peritonitis (SBP) and sepsis]
  6. Need for paracentesis
  7. Multiorgan failure
  8. Acute-on-chronic liver failure (ACLF)
- Continuous outcomes
  1. Renal impairment
    - Serum creatinine (in mg/dL)—as a measure of renal impairment
    - Serum sodium (in mEq/L)—as a measure of hyponatremia
  2. Plasma renin activity (PRA) (in ng/mL/h)—as a measure of PICD/circulatory dysfunction.

These endpoints are collected at the end of the study/at follow-up.

### Data extraction

We exported the searches to a reference management software (Endnote 21.1) and removed duplicate records. Studies that fit the inclusion criteria were screened by title and abstract by 2 reviewers (Reem M. AlSowaiegh, Alastair O’Brien). Uncertainty and disagreement were resolved by discussion and, if required, by consulting a third reviewer (Nick Freemantle).

Full-text article availability (and eligible abstracts) extraction of data elements was performed by 2 review authors (Reem M. AlSowaiegh, Khalifa A. Yusuf) using a customized electronic Excel data extraction form. The main data fields were study author, study year, study country, sample size, duration of follow-up, intervention (intravenous HAS), alternative therapies, HAS indication [including Large Volume Paracentesis (LVP), long-term HAS, spontaneous bacterial peritonitis (SBP), non-SBP, HE, sepsis-induced hypotension, and hospitalizations with complications of liver cirrhosis] and outcomes (complications of liver cirrhosis) (Table 1). The outcomes data were collected from tables and texts of the RCTs. The authors did not assess various definitions of each endpoint in each RCT. The main data elements (indications for HAS and outcomes reported at the end of the study) were then reverified by another independent reviewer (Alastair O’Brien). Any uncertainties or disagreements over data extraction elements' eligibility were resolved by discussion, and if required, by consulting a third reviewer (Nick Freemantle).

**TABLE 1:** Characteristics of studies table

Study author	Year	Country	Sample size	IV HAS	Alternatives	HAS indication	Outcomes	Follow-up duration
Abdel-Khalek	2010	Egypt	135	20% HAS (8 g/L ascitic fluid removed)	HES 6%	LVP	Mortality, renal impairment, Sr. Cr, hyponatremia, Sr. Na, HE, GI bleeding, ascites recurrence, non-SBP, PICD, and PRA	6 mo
Abootalebi	2017	Iran	108	20% HAS (5 g/L of paracentesis fluid)	HES 6% with 5 g/L of parasynthesized liquid	LVP	Sr. Na	4 d
Alessandria	2011	Italy	70	20 g of HAS (8 g/L of ascites removed)	20 g of HAS 20 g (4 g/L of ascites removed)	LVP	Mortality, renal impairment, Sr. Cr, hyponatremia, Sr. Na, HE, GI bleeding, ascites recurrence, SBP, non-SBP, PICD, PRA, and portal hypertensive bleeding	6 mo
AlSebaey	2013	Egypt	125	20% low-dose HAS (2 g/L) and 20% standard-dose HAS (6 g/L)	IV terlipressin 3 mg vs. IV HES 6% (8 g/L removed) vs. oral midodrine (5–10 mg three times daily)	LVP	Sr. Na and PICD	6 d
Altman	1998	France	60	20% HAS (8 g/L of ascites removed)	6.5% HES (200 mL/L of ascites removed)	LVP	Renal impairment, Sr. Cr, hyponatremia, Sr. Na, HE, GI bleeding, ascites recurrence, non-SBP, and need for paracentesis	15 d
Appenrodt	2008	Germany	24	HAS (8 g/L of ascites removed)	Midodrine (12.5 mg every 8 h for 2 d)	LVP	Mortality, renal impairment Sr. Cr, hyponatremia, Sr. Na, HRS, and PICD	6 d
Arora	2020	India	80	20% HAS (8 g/dL of ascitic fluid removed)	No HAS	LVP	Mortality, renal impairment, Sr. Cr, hyponatremia, Sr. Na, HE, non-SBP, PICD, and PRA	28 d
Baik	2000	South Korea	23	HAS (6 g/L of ascites removed)	No HAS	LVP	Sr. Cr, HE, and PRA	48 h
Bari	2012	United States	25	HAS (8 g/L of ascites removed)	10 mg oral midodrine (3 times daily) and IM 20 μg octreotide	LVP	Mortality, renal impairment, Sr. Cr, Sr. Na, HE, GI bleeding, HRS, PICD, PRA, and need for paracentesis	10 mo
Bertran	1991	Spain	17	HAS (8 g/L of extracted ascitic fluid) and total paracentesis	IV dextran-70 (8 g/L of extracted ascitic fluid) and total paracentesis	LVP	Mortality, renal impairment, hyponatremia, and HE	1 mo
Bruno	1992	Italy	35	HAS and total paracentesis (4–6 g of ascites removed)	Spontaneous ascites filtration and reinfusion (SAFR)	LVP	Mortality, hyponatremia, and Sr. Na	8 d
Caraceni (ANSWER Trial)	2018	Italy	431	SMT and 20% HAS (40 g twice weekly for 2 wk then 40 g weekly)	SMT	Long-term HAS administration	Mortality, renal impairment, GI bleeding, ascites recurrence, HRS, non-SBP, ACLF, portal hypertensive bleeding, and need for paracentesis	18 mo
Chen	2009	Taiwan	30	20% HAS (50 mL for 3 d) + antibiotics (cephalosporins)	Antibiotic (cephalosporins)	SBP	Mortality, renal impairment, Sr. Cr, and Sr. Na	6 d
China (ATTIRE Trial)	2021	United Kingdom	777	20% HAS for up to 14 d	SMT	HAS to prevent infection, renal dysfunction, and death	Mortality, renal impairment, HE, GI bleeding, ascites recurrence, SBP, non-SBP, multiorgan failure, PICD, and portal hypertensive bleeding	6 mo
Degoricija	2003	Croatia	50	200 mL of 20% HAS and paracentesis (6.6 g/L ascites removed)	Fresh frozen plasma (600 mL), haemaccel (900 mL), spironolactone (100 mg), and furosemide (40 mg)	LVP	Mortality and Sr. Cr	6 d
Devisetty	2024	India	174	HAS 20 g/d × 5 d	HAS (1.5 g/kg on day 1, 1 g/kg on day 3)	Non-SBP	Mortality and Sr. Cr	30 d
Fagan (HEAL Trial)	2023	United States	48	25% HAS 1.5 g/kg once a week for 5 wk	Normal saline (placebo) once a week for 5 wk	HE	Sr. Na, HE, and non-SBP	6 wk
Fassio	1992	Argentina	41	20% HAS with paracentesis (6 g for each 1000 mL of ascites removed)	Dextran-70 (5 L) with paracentesis (6 g for each 1000 mL of ascites removed)	LVP	Mortality, renal impairment, Sr. Cr, hyponatremia, Sr. Na, HE, GI bleeding, ascites recurrence, HRS, non-SBP, and PRA	32 wk
Fernandez	2005	Spain	20	20% HAS 1.5 g/kg after baseline and 1 g/kg on day 3	6% HES (200/0.5) 1.5 g/kg after baseline and 1 g/kg on day 3	SBP	Renal impairment, Sr. Cr, Sr. Na, HRS, PICD, and PRA	4 d
Fernandez (INFECIR-2 Trial)	2020	Spain	118	20% HAS 1.5 g/kg on day 1 and 1 g/kg on day 3, and antibiotics	Antibiotics alone	Non-SBP	Mortality, renal impairment, Sr. Cr, Sr. Na, GI bleeding, SBP, non-SBP, ACLF, and portal hypertensive bleeding	90 d
Garcia-Compean	1993	Mexico	35	HAS (5 g/L of removed ascites) with TTP	TTP without HAS	LVP	Mortality, renal impairment, Sr. Cr, hyponatremia, Sr. Na, and HE	5 d
Garcia-Compean	2002	Mexico	96	HAS (8 g/L of ascites removed) and LVP	IV Dextran-40 (8 g/L of ascites removed) with LVP	LVP	Mortality, renal impairment, Sr. Cr, hyponatremia, Sr. Na, GI bleeding, ascites recurrence, HRS, non-SBP, PICD, and PRA	44 mo
Gentilini Protocol 1	1999	Italy	126	HAS 12.5 g/d and diuretics	Diuretics alone	Long-term HAS	Mortality, renal impairment, hyponatremia, Sr. Na, HE, ascites recurrence, and portal hypertensive bleeding	24 d
Gentilini Protocol 2	1999	Italy	81	HAS 25 g/wk and diuretics	Diuretics and low low-sodium diet	Long-term HAS	Mortality, renal impairment, HE, GI bleeding, ascites recurrence, and SBP	3 y
Gines	1987	Spain	117	HAS 40 g and paracentesis (4–6 L/d ascites removal)	Spironolactone (200–400 mg/d) and furosemide (40–240 mg/d)	LVP	Mortality, renal impairment, Sr. Cr, hyponatremia, Sr. Na, HE, GI bleeding, ascites recurrence, SBP, and non-SBP	47 wk
Gines	1988	Spain	105	20% HAS 40 g and paracentesis (4–6 L/d ascites removal)	Paracentesis (4–6 L/d ascites removal) without HAS	LVP	Mortality, renal impairment, Sr. Cr, hyponatremia, Sr. Na, HE, GI bleeding, ascites recurrence, SBP, non-SBP, and PRA	40 wk
Gines	1996	Spain	289	20% HAS (8 g/L of ascitic fluid removal) and total paracentesis	Total paracentesis and IV dextran-70 (8 g/L of ascitic fluid removal) and IV polygeline (8 g/L of ascitic fluid removal)	LVP	Mortality, renal impairment, Sr. Cr, hyponatremia, Sr. Na, HE, GI bleeding, non-SBP, PICD, and PRA	56 mo
Guevara	2012	Spain	97	20% HAS (1.5 g/kg on day 1, then 1 g/kg on day 3) and antibiotics	Antibiotics alone	Non-SBP	Mortality, renal impairment, Sr. Cr, HRS, and PRA	3 mo
Hamdy	2014	Egypt	50	HAS (8 g/L of ascitic fluid removal)	Oral midodrine 12.5 mg every 8 h for 3 d	LVP	Mortality, renal impairment, Sr. Cr, Sr. Na, HE, GI bleeding, HRS, and PRA	19 d
Hernandez-Perez	1995	Mexico	16	20% HAS (6 g/L of extracted fluid)	Dextran-70 (6 g/L of extracted fluid)	LVP	Mortality, renal impairment, Sr. Cr, hyponatremia, Sr. Na, ascites recurrence, and PRA	2 wk
Jalan (Abstract)	2007	United Kingdom	24	HAS 40 (g/day) for 7 d + fluid restriction (1.5 L) + sodium restriction (80 mmol/d)	Fluid restriction (1.5 L) + sodium restriction (80 mmol/d)	LVP	Mortality, renal impairment, HE, and non-SBP	7 d
Jha	2023	India	50	HAS after LVP	Polygeline infusion after LVP	LVP	Renal impairment and Sr. Cr	6 d
Kang	1998	South Korea	12	HAS (8 g/L ascites removed)	HES	LVP	Mortality, renal impairment, Sr. Cr, hyponatremia, Sr. Na, and HE	3 d
Kar	2023	India	63	20% HAS (1.5 g/kg on day 1, then 1 g/kg on day 3)	Reduced dose 20% HAS (0.75 g/kg day 1 then 0.5 g/kg on day 3)	SBP	Mortality, renal impairment, Sr. Cr, and SBP	28 d
Khan	2015	Pakistan	50	HAS (6 g/L of ascitic fluid removed)	Polygelene (haemaccel) (125 mL/L of ascites removed)	LVP	Mortality, Sr. Cr, Sr. Na, and ascites recurrence	6 d
Lata	2007	Czech Republic	49	20% HAS (8 g/L of removed ascites)	IV terlipressin (1 mg every 4 h for 48 h)	LVP	HE and PRA	7 d
Luca	1995	Spain	18	20% HAS (8 g/L of ascites removed)	No HAS	LVP	Mortality, Sr. Cr, Sr. Na, and PRA	24 Hours
Maiwall (ALPS Trial)	2022	India	100	20% HAS (0.5–1 g/kg over 3 h)	Plasmalyte-148 solution (30 mL/kg over 3 h)	Sepsis-induced hypotension	Mortality, renal impairment, and Sr. Cr	28 d
Maiwall (Abstract)	2023	India	106	20% HAS (60 g/week for 2 wk then 20–40 g/wk for 12 mo) and SMT	SMT	Long-term HAS	Mortality, renal impairment, HE, and non-SBP	12 mo
Maiwall (Abstract)	2024	India	121	20% HAS with SMT	SMT	Long-term HAS	Mortality, renal impairment, and HE	12 mo
Moreau	2002	France	20	20% HAS (8 g/L of removed ascites) and paracentesis	IV terlipressin 3 mg and paracentesis	LVP	Mortality, renal impairment, Sr. Cr, hyponatremia, Sr. Na	3 mo
Moreau	2006	France	68	20% HAS	3.5% polygeline (haemaccel)	LVP	Mortality, renal impairment, hyponatremia, HE, ascites recurrence, SBP, non-SBP, and portal hypertensive bleeding	6 mo
Naidu (Abstract)	2024	India	66	Standard dose 20% HAS (8 g/L of ascitic fluid drained)	Low dose 20% HAS (4 g/L of ascitic fluid drained)	LVP	Renal impairment, hyponatremia, and ascites recurrence	6 d
O’Leary (PRECIOSA Trial) (Abstract)	2025	United States	410	20% HAS (1.5 g/kg every 10 d ±2 d for 12 mo) and SMT	SMT	Long-term HAS	Mortality	12 mo
Philips (FRISC Trial)	2021	India	308	5% HAS (250 mL bolus then 50 mL/h for 3 h	0.9% normal saline (30 mL/kg bolus then 100 mL/h for 3 h	Sepsis-induced hypotension	Mortality	1 wk
Planas	1990	Spain	88	20% HAS (8 g/L of ascites removed) and total paracentesis	IV dextran-70 (8 g/L of ascites removed) and total paracentesis	LVP	Mortality, renal impairment, Sr. Cr, hyponatremia, Sr. Na, HE, GI bleeding, ascites recurrence, SBP, non-SBP, PRA, and portal hypertensive bleeding	27 wk
Reddy	2023	India	50	20% HAS (100 mL over 4 h)	Oral midodrine hydrochloride 7.5 mg 3 times a day for 3 d	LVP	Mortality, renal impairment, Sr. Cr, hyponatremia, Sr. Na, HE, GI bleeding, non-SBP, multiorgan failure, PICA, and PRA	28 d
Romanelli	2006	Italy	100	HAS (25 g/wk for 1 y then 25 g every 2 wk) and diuretics	Diuretics alone (spironolactone 100–400 mg and furosemide 25–150 mg)	Long-term HAS	Mortality, HE, ascites recurrence, HRS, SBP, non-SBP, ACLF, and portal hypertensive bleeding	120 mo
Salerno	1987	Italy	41	HAS (20–60 g after paracentesis) and paracentesis (4 L/d)	Diuretics (spironolactone 200 mg/d and furosemide 50 mg/d)	LVP	Mortality, renal impairment, Sr. Na, HE, GI bleeding, ascites recurrence, and PRA	19 wk
Salerno	1991	Italy	54	20% HAS (30 mL/L ascites removed) and paracentesis	IV haemaccel (150 mL/L ascites removed) and paracentesis	LVP	Mortality, renal impairment, Sr. Cr, hyponatremia, Sr. Na, HE, GI bleeding, ascites recurrence, SBP, non-SBP, and PRA	63 wk
Salman	2016	Egypt	200	20% HAS (1.5 g/kg day 1 then 1 g/kg on day 3) and low-dose albumin (10 g/12 h) and IV terlipressin (1 mg/6 h)	IV terlipressin (1 mg/6 h for 3 d) and oral midodrine (10 mg/8 h for 10 d)	SBP	Mortality, renal impairment, SBP, and PRA	1 mo
Sharma	2017	India	120	HAS (1.5 g/kg/day) and lactulose (30–60 mL 3 times a day)	Lactulose 30–60 mL 3 times a day	HE	Mortality, HE, GI bleeding, HRS, SBP, and non-SBP	10 d
Simon-Talero	2013	Spain	56	20% HAS (1.5 g/kg on day 1, then 1 g/kg on day 3)	Isotonic saline (0.9%)	HE	Mortality, HE, GI bleeding, and multiorgan failure	90 d
Singh (Noradrenaline Study)	2006	India	40	HAS (8 g/L of ascitic fluid removed)	IV noradrenaline (0.5 mg/h)	LVP	Renal impairment, Sr. Cr, hyponatremia, Sr. Na, PICD, and PRA	6 d
Singh (Terlipressin Study)	2006	India	40	HAS (8 g/L of ascitic fluid removed)	IV terlipressin (3–1 mg bolus then at 8and 16 h)	LVP	Renal impairment, Sr. Cr, hyponatremia, Sr. Na, PICD, PRA, and need for paracentesis	3 mo
Singh	2008	India	40	HAS (8 g/L of ascitic fluid removed)	Oral midodrine (5–10 mg 8 hourly for 72 h)	LVP	Mortality, renal impairment, Sr. Cr, hyponatremia, Sr. Na, GI bleeding, SBP, PICD, PRA, and need for paracentesis	3 mo
Sola (MACHT Trial)	2018	Spain	173	20% HAS (40 g/15 d) and oral midodrine (15–30 mg/d)	Placebo	Long-term HAS	Mortality, renal impairment, Sr. Cr, hyponatremia, Sr. Na, HE, GI bleeding, non-SBP, and multiorgan failure	12 mo
Sola-Vera	2003	Spain	72	20% HAS (8 g/L of ascites removed)	IV saline (170 mL/L of 3.5% saline solution per liter of ascites removed at 999 mL/h)	LVP	Mortality, renal impairment, Sr. Cr, hyponatremia, Sr. Na, GI bleeding, SBP, non-SBP, PICD, PRA, and portal hypertensive bleeding	1 mo
Sort	1999	Spain	126	20% HAS (1.5 g/kg on day 1, then 1 g/kg on day 3) and cefotaxime	IV cefotaxime (2 g every 6 h, 1 g every 6 h, 1 g every 8 h, and 1 g every 12 h)	SBP	Mortality, renal impairment, Sr. Cr, Sr. Na, ascites recurrence, SBP, non-SBP, PRA, and need for paracentesis	3 mo
Surabhi (Abstract)	2024	India	50	HAS (25 g/wk for the first year, then 25 g every 2 weeks) and diuretics	Diuretics alone	Long-term HAS	Ascites recurrence	1 y
Tarao	1983	Japan	17	Salt-poor HAS	Diuretics (furosemide or spironolactone)	Long-term HAS	Mortality and ascites recurrence	2 y
Thevenot	2015	France	191	20% HAS (1.5 g/kg on day 1, then 1 g/kg on day 3) and antibiotics	Antibiotics alone	Non-SBP	Mortality, renal impairment, Sr. Cr, GI bleeding, multiorgan failure, and portal hypertensive bleeding	3 mo
Ventura-Cots (BETA Trial)	2021	Spain	82	HAS (1.5 g/kg day 1 then 1 g/kg day 3)	Placebo (100 ml of 0.9% saline solution)	Hepatic encephalopathy	Mortality, hyponatremia, HE, GI bleed, ascites recurrence, SBP, and non-SBP	180 d
Wilkinson	1962	United Kingdom	16	Salt-poor HAS with pyrogen-free water to a 25% solution	SMT	Long-term HAS	Mortality, ascites recurrence, and portal hypertensive bleeding	22 mo
Xue	2002	China	112	HAS (0.5–1.0 g/kg within 6 h on day 3, then every 3 d for 3 wk) and IV ceftriaxone	IV ceftriaxone (3 g/d)	SBP	Mortality, renal impairment, GI bleeding	3 wk
Yosry	2019	Egypt	75	HAS (8 g/L of removed ascitic fluid)	Oral midodrine for 2 d (12.5 mg) and oral midodrine for 30 d (12.5 mg)	LVP	Mortality, renal impairment, Sr. Cr, hyponatremia, Sr. Na, HE, and ascites recurrence	30 d
Zaak	2001	Germany	35	HAS (5 g/L of ascites removed)	IV reinfusion of ultrafiltrate-ascitic fluid by hemofiltration	LVP	Mortality, renal impairment, Sr. Cr, hyponatremia, HE, GI bleeding, SBP, and PRA	28 mo
Zhao	2000	China	68	HAS (20 g given when 3000–600 mL of ascites removed)	IV 20% mannitolum (50 g given when 3000–600 mL of ascites removed)	LVP	Mortality, HE, GI bleeding, ascites recurrence, and SBP	30 wk

Abbreviations: HE, hepatic encephalopathy; HES, hydroxyethyl starch; HRS, hepatorenal syndrome; LVP, large volume paracentesis; PRA, plasma renin activity; SBP, spontaneous bacterial peritonitis; SMT, standard medical treatment; Sr. Cr, serum creatinine; Sr. Na, serum sodium; TTP, total therapeutic paracentesis.

The study selection process and reasons for excluding ineligible studies are presented in the PRISMA 2020 reference flow diagram (Figure 1) and Table 1 presents the characteristics of all RCTs included in the systematic review.

**FIGURE 1:**
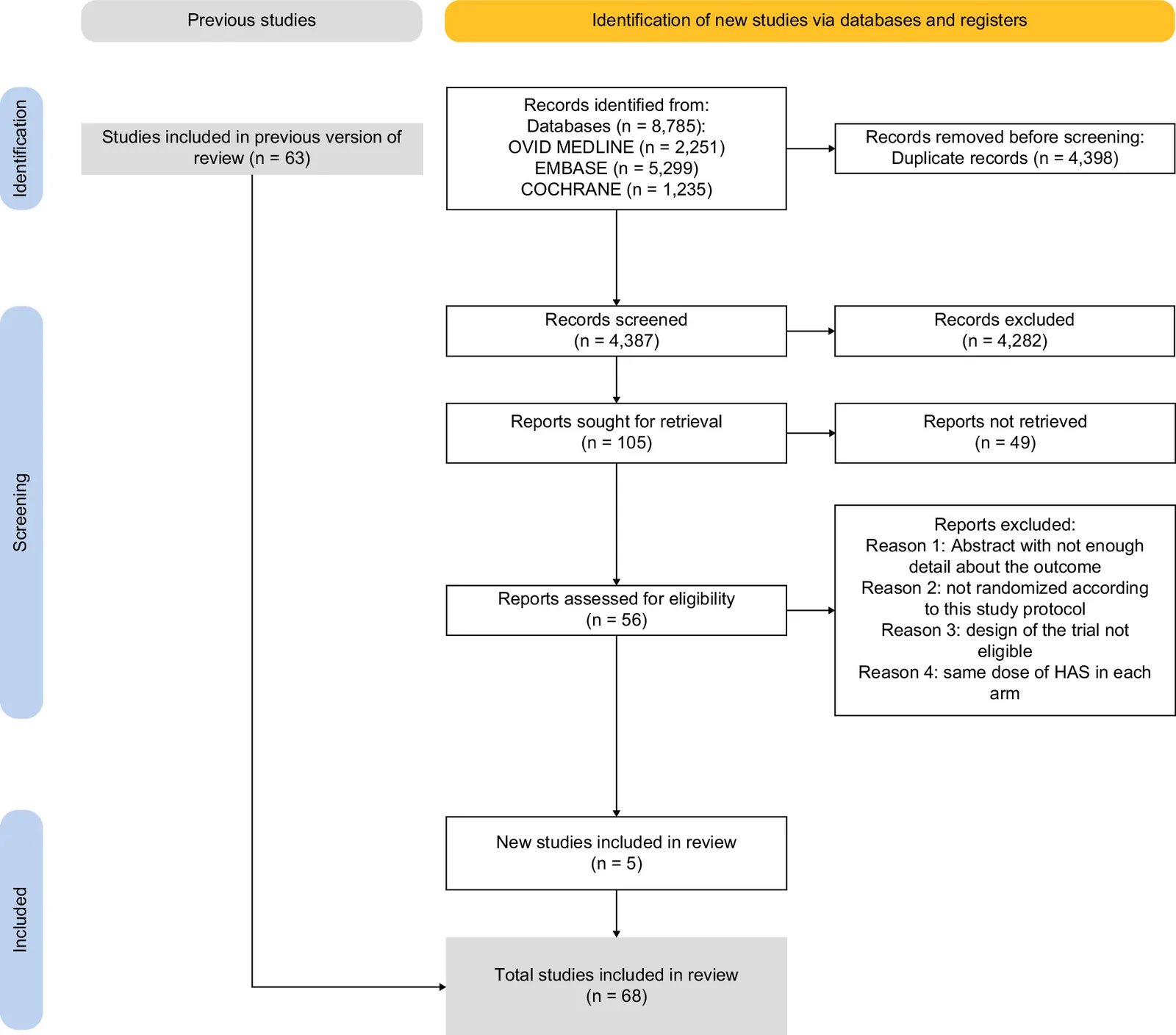
PRISMA 2020 reference flow diagram for updated systematic reviews, which included searches of databases and registers only. Abbreviations: HAS, human albumin solution; PRISMA, Preferred Reporting Items for Systematic Reviews and Meta-Analyses.

### Risk of bias (quality) assessment

We used the validated Cochrane Collaboration's “Risk of Bias assessment tool 2” (RoB2) to assess risk of bias for mortality as an outcome. In all, 56/68 studies reported mortality. These studies were assessed using the standardized RoB2 form by 2 independent review authors (Reem M. AlSowaiegh, Khalifa A. Yusuf) for the randomization process, deviations from the intended interventions, missing outcome data, measurement of outcome, and selection of the reported result. The final assessment of studies is presented as a traffic light figure (Figure 2) that shows the level of bias in the different domains of individual studies and across all the RCTs in the review.

**FIGURE 2:**
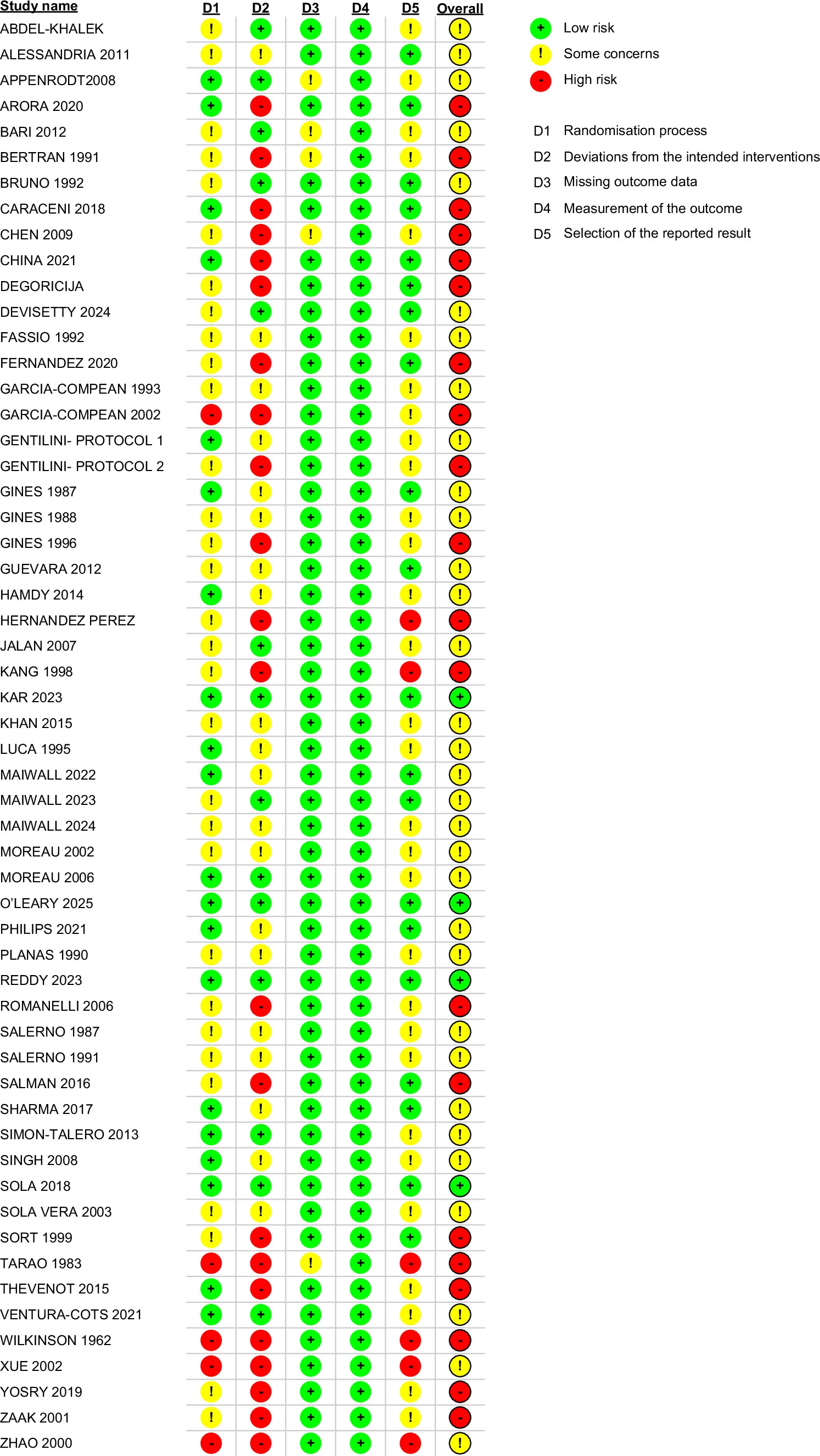
Risk of bias.

### Statistical analysis

The meta-analysis was performed by StatsDirect software version 4.0.4. Outcomes were analyzed at the level of individual liver cirrhosis-related complications within the studies, and subgroup analyses were based on sample size.

Dichotomous outcomes are expressed as random effect odds ratios (ORs) with 95% confidence intervals (CIs). *p*-value <0.05 was considered statistically significant. For the overall analyses, we used the DerSimonian–Laird method to estimate the random-effects pooled OR. The exact mid-P conditional maximum likelihood method was used to estimate the fixed-effects pooled ORs for each stratum. Continuous outcomes (serum creatinine, serum sodium, and plasma renin activity) are expressed as mean ± standard deviation and pooled using a weighted mean difference. The Mulrow–Oxman method was used to estimate the fixed-effects pooled weighted mean difference, and the DerSimonian–Laird method was used to estimate the random pooled weighted mean difference.

We investigated the potential sources of statistical heterogeneity. We used forest plots, the Cochran *Q* test, and the Higgins *I* ^2^ statistic to assess the statistical heterogeneity. We also performed a qualitative assessment of bias indicators, including the Egger test, Harbord–Egger test, and the Begg–Mazumdar test.

Publication bias may be more likely for smaller trials, and so we performed a subgroup analysis to explore sources of clinical or methodological heterogeneity. We divided the trials into large (sample size ≥200), medium (sample size 101–199), and small trials (sample size ≤100) that reported mortality and renal impairment as outcomes. A cutoff sample size of 100 was chosen after determining that 102 was the overall mean of sample sizes for studies that reported mortality. This resulted in a small number of large trials. Yet, publishing the findings of these large trials is important to decrease publication bias. Additional sensitivity analysis was performed by dividing the studies that reported mortality into inpatient studies and outpatient studies to consider albumin’s significance regarding mortality in inpatients versus outpatients.

## RESULTS

### Search results

The PRISMA 2020 reference flow diagram (Figure 1) started with 8785 studies from 3 databases: Medline, Embase, and Cochrane. Duplicates were removed, leaving 4398. These were manually screened for eligibility by reading the title and abstract. This resulted in 105 eligible studies. After retrieving the full-text, only 56 fit the inclusion criteria. We identified 2 recent HAS systematic reviews^6^^,^^7^ and found an extra 16 studies within them. After extracting these 16 articles and reading the full text, only 12 fit our eligibility criteria, resulting in 68 included studies. The 68 studies included 62 full-text articles and 6 abstracts.

The 4293 excluded studies were mainly due to their design, with studies not randomized, or the same dose of HAS given in each arm, or the abstract not having enough detail on outcomes to analyze for meta-analysis purposes.

### Characteristics of included studies

The characteristics of the 68 RCTs in the systematic review are presented in Table 1. These included 6714 patients from various countries. The alternative treatments to HAS were hydroxyethyl starch (HES), vasopressors, fluids, antibiotics, diuretics, standard medical treatment (SMT), or no HAS infusion. A list of outcomes reported in each study is included in the table.

### Risk of bias

The risk of bias for the 56 studies that reported our primary outcome, mortality, is shown in Figure 2 using the Cochrane Collaboration RoB2 tool. Four studies were considered to have low risk of bias (Kar et al.,^12^ O’Leary et al.,^13^ Reddy et al.,^14^ and Sola et al.^15^), 32 studies were considered to have some risk of bias, and 20 studies were considered to have high risk.

### Effect measures/meta-analysis

A summary of the outcomes is presented in Table 2 and Figure 3.

**TABLE 2:** Summary of reported findings

Outcomes	Number of studies with reported outcome	Overall pooled effect estimate	95% confidence interval (CI)	*p*
Mortality (OR)	56 studies	0.769	0.652–0.908	0.0019
Renal dysfunction				
Renal impairment (OR, mean difference)	Binary outcome: 48 studies	0.560	0.426–0.735	<0.0001
	Continuous outcome (serum creatinine): 40 studies	−0.036	−0.093 to 0.021	0.2179
Hyponatremia (OR, mean difference)	Binary outcome: 30 studies	0.524	0.395–0.696	<0.0001
	Continuous outcome (serum sodium): 37 studies	1.474	0.645–2.304	0.0005
Hepatorenal syndrome (HRS) (OR)	10 studies	0.347	0.145–0.831	0.0175
Paracentesis-induced circulatory dysfunction (PICD) (OR, mean difference)	Binary outcome: 15 studies	0.543	0.351–0.840	0.006
	Continuous outcome (plasma renin activity): 26 studies	−1.568	−2.606 to −0.529	0.003
Hepatic encephalopathy (HE)(OR)	35 studies	0.806	0.619–1.048	0.1075
Gastrointestinal bleeding (OR)	28 studies	0.947	0.679–1.321	0.7497
Portal hypertensive bleeding (OR)	11 studies	1.11	0.74–1.66	0.6229
Recurrence of ascites (OR)	26 studies	0.694	0.502–0.959	0.0267
Development of bacterial infection (OR)	SBP: 19 studies	0.749	0.472–1.190	0.2215
	Non-SBP (including sepsis): 25 studies	0.887	0.647–1.215	0.4546
Need for paracentesis (OR)	6 studies	0.59	0.34–1.03	0.0656
Multiorgan failure (OR)	5 studies	0.79	0.49–1.28	0.3481
Acute-on-chronic liver failure (ACLF) (OR)	3 studies	0.66	0.37–1.18	0.1643

**FIGURE 3:**
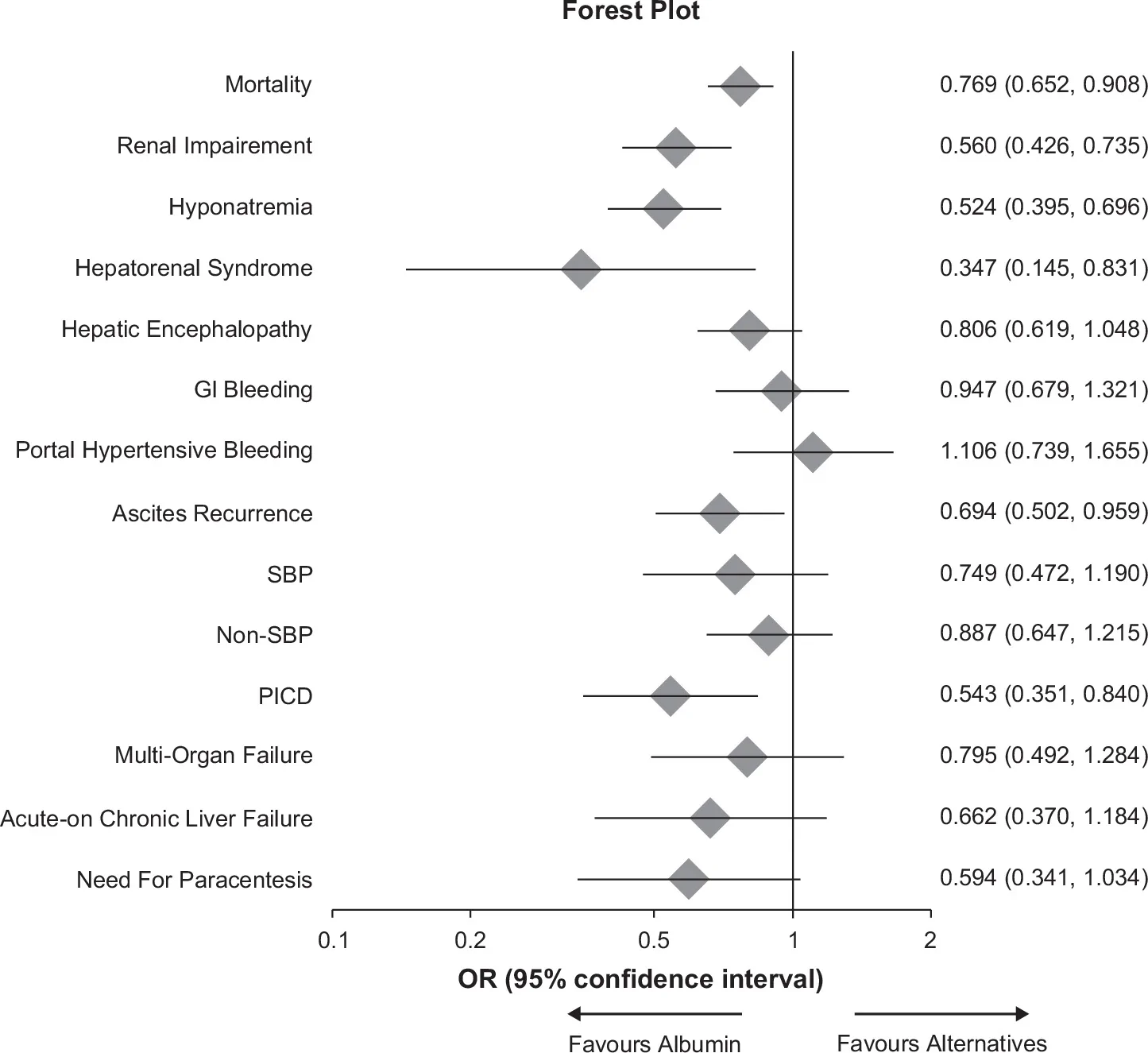
Summary of all outcomes of liver cirrhosis complications (OR, 95% CI). Abbreviations: PICD, paracentesis-induced circulatory dysfunction; SBP, spontaneous bacterial peritonitis.

#### Mortality

The 56 studies that reported mortality showed a reduction (23.1%) in the overall odds of death compared with alternatives, with overall pooled random OR 0.769 (95% CI 0.652–0.908; *p*=0.0019). HAS had the greatest effect on reducing mortality when used for SBP and HE; OR 0.497 (95% CI 0.328–0.754; *p*=0.001) and OR 0.419 (95% CI 0.232–0.756; *p*=0.0039), respectively (Figure 4).

**FIGURE 4:**
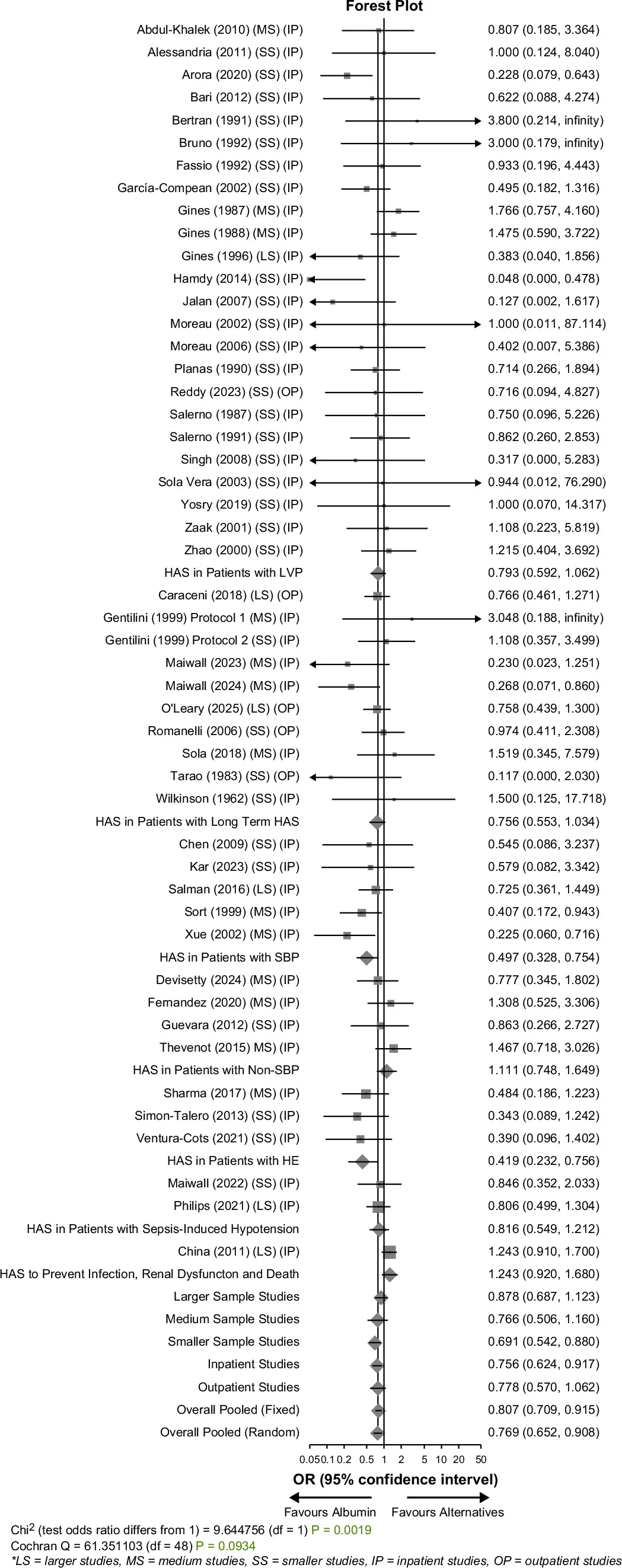
Mortality reported in all trials (grouped according to clinical indication for HAS use in liver cirrhosis) (OR, 95% CI). Abbreviations: HAS, human albumin solution; LVP, large volume paracentesis; SBP, spontaneous bacterial peritonitis.

Subgroup analyses of trials according to size showed larger studies (≥200) had a 12.2% reduction in the overall odds of death, OR 0.878 (95% CI 0.687–1.123; *p*=0.3008). Medium studies (101–199) had a 23% reduction in overall odds of death, OR 0.77 (95% CI 0.51–1.16; *p*=0.2082). Smaller trials (≤100) demonstrated a 31.3% reduction in the overall odds of death, OR 0.687 (95% CI 0.540–0.875; *p*=0.0023). In addition, sensitivity analysis was done to compare the effect of HAS in inpatient and outpatient studies. The overall random OR for hospitalized patients given HAS was 0.756 (95% CI 0.624–0.917; *p*=0.004), the overall random OR for outpatients was 0.778 (95% CI 0.570–1.062; *p*=0.1138) (Figure 4).

Reporting bias for mortality as an outcome produced a degree of funnel plot asymmetry around the effect estimate. This was also indicated by the reporting bias indicators: Begg–Mazumdar, Egger, and Harbord–Egger. Measures of heterogeneity were Cochran *Q* = 61.351103 (df = 48) *p*=0.0934, *I* ^2^ (inconsistency) = 21.8%, Chi^2^ (test odds ratio differs from 1) = 9.644756 (df = 1) *p*=0.00219 (Supplemental Materials, http://links.lww.com/HC9/C224).

#### Renal dysfunction

- Renal impairment

Forty-eight studies reported renal impairment. There was a 44% reduction in overall odds of renal impairment with the use of HAS compared with alternatives, OR 0.560 (95% CI 0.426–0.735; *p*<0.0001). HAS reduced renal impairment in large trials (OR 0.675, 95% CI 0.480–0.949; *p*=0.0236), medium trials (OR 0.40, 95% CI 0.21–0.76; *p*=0.0049), and small trials (OR=0.631, 95% CI 0.445–0.896; *p*=0.0099). A greater beneficial effect was seen in inpatients (OR 0.552, 95% CI 0.420–0.724; *p*<0.0001). HAS was most beneficial in reducing renal impairment when used for LVP and SBP, OR 0.589 (95% CI 0.379–0.916; *p*=0.0187) and OR 0.361 (95% CI 0.224–0.581; *p*<0.0001), respectively (Figure 5A).

B. Serum creatinine

FIGURE 5(A) Renal impairment reported in all trials (grouped according to clinical Indication for HAS use in liver cirrhosis) (OR, 95% CI). (B) Hyponatremia was reported in all trials (grouped according to clinical Indication for HAS use in liver cirrhosis) (OR, 95% CI). (C) Hepatorenal syndrome was reported in all trials (grouped according to clinical Indication for HAS use in liver cirrhosis) (OR, 95% CI). (D) Paracentesis-induced circulatory dysfunction was reported in all trials (grouped according to clinical Indication for HAS use in liver cirrhosis) (OR, 95% CI). Abbreviations: HAS, human albumin solution; LVP, large volume paracentesis; SBP, spontaneous bacterial peritonitis.

**Figure:**
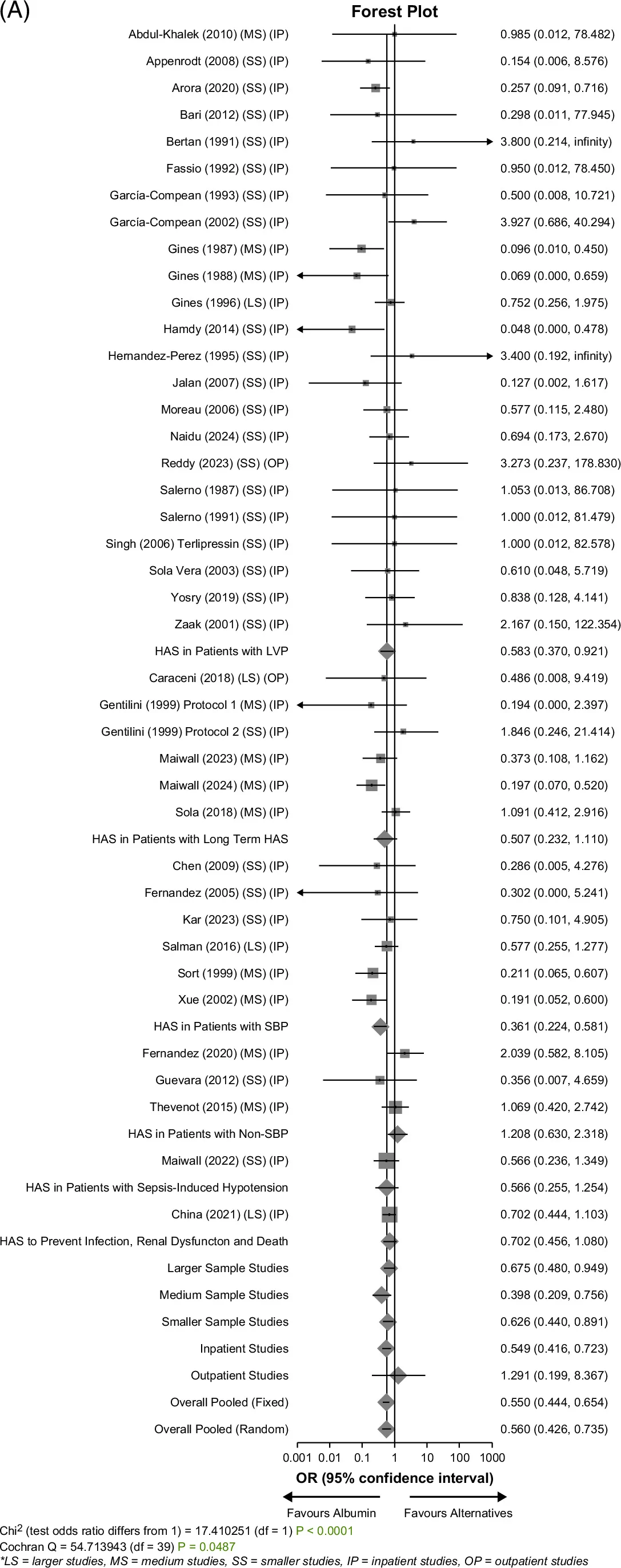

**Figure:**
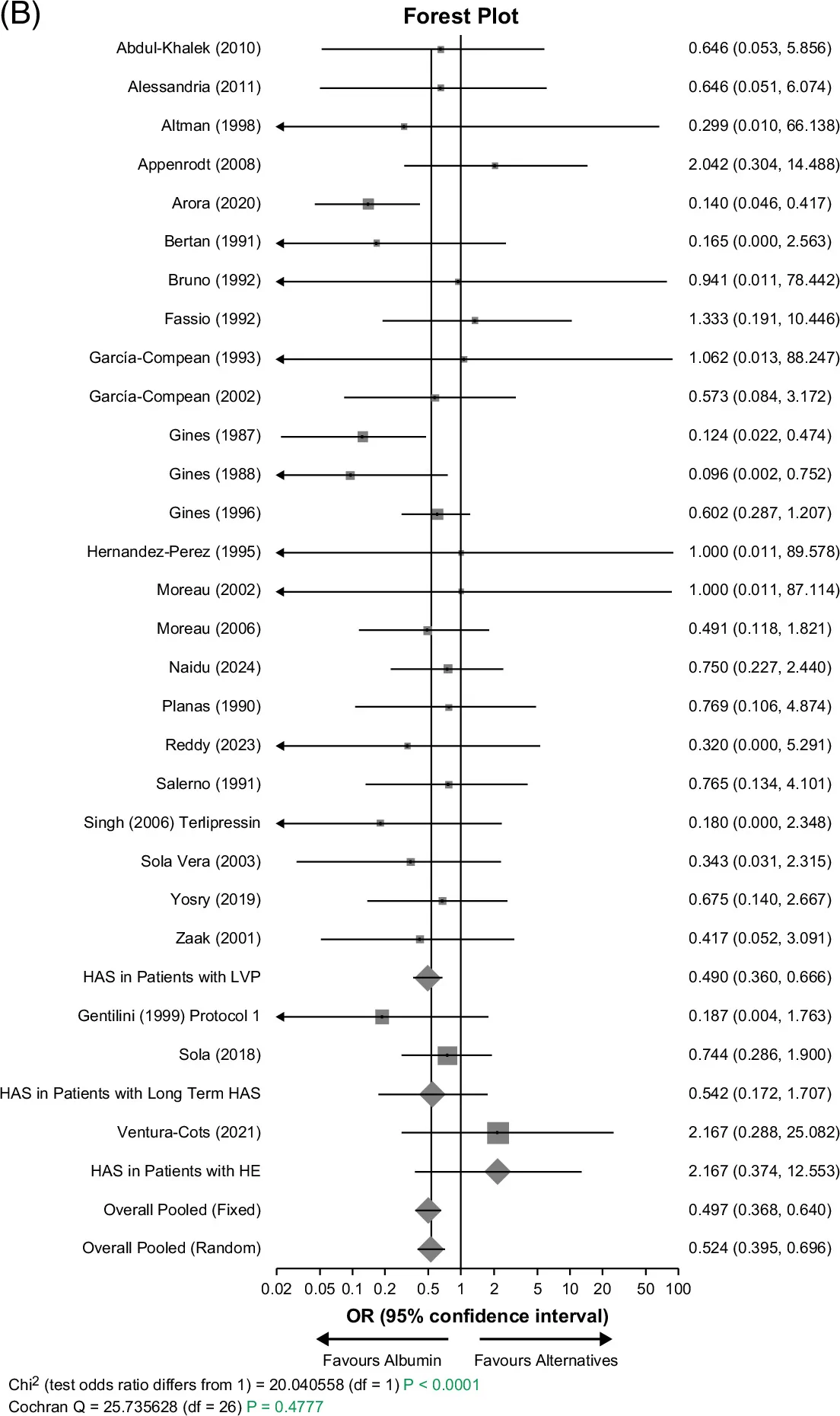

**Figure:**
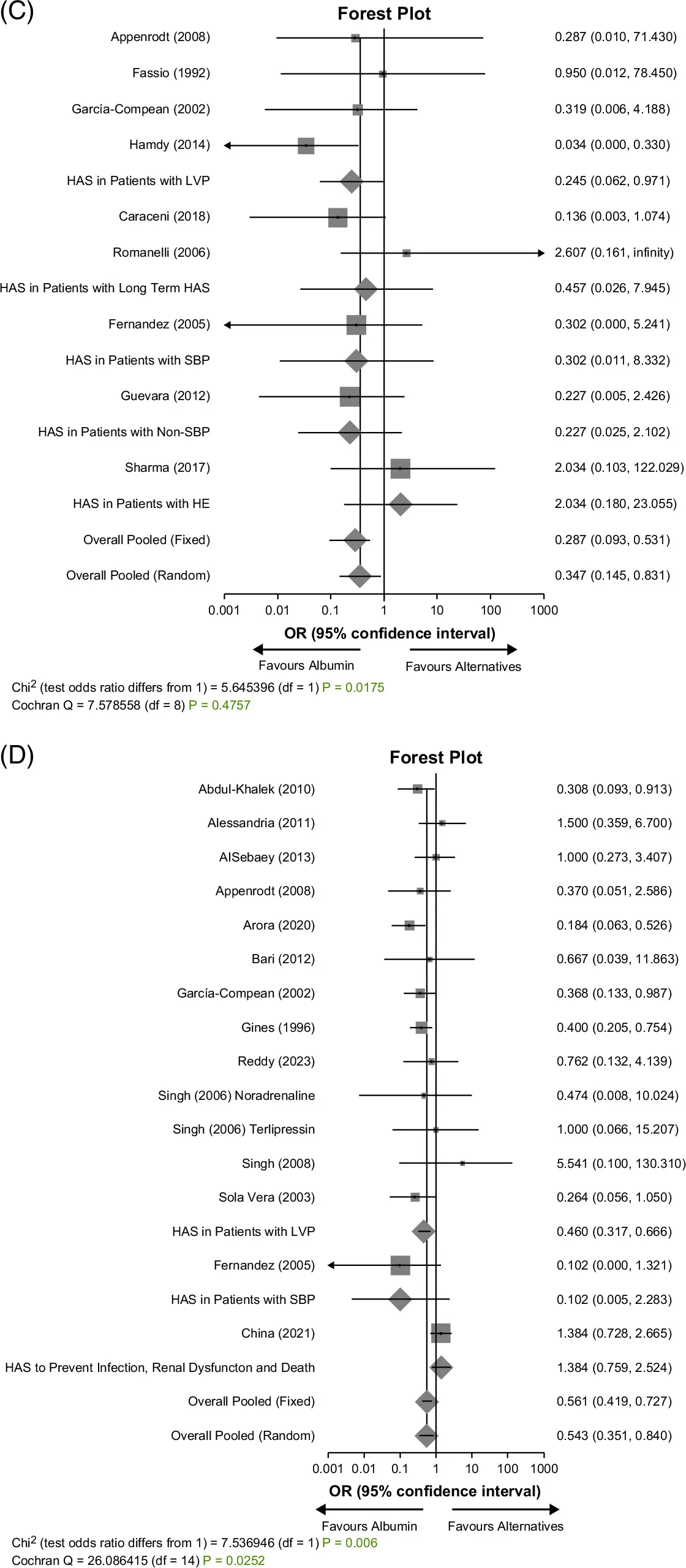

Forty studies reported serum creatinine as a measure of renal impairment, and this was not reduced by use of HAS compared with alternatives, with a pooled weighted mean difference (WMD) of −0.036 (95% CI −0.093 to 0.021; *p*=0.2179) (Supplemental Materials, http://links.lww.com/HC9/C224).

C. Hyponatremia

Hyponatremia was reported in 30 studies. There was a 47.6% reduction in the overall odds of hyponatremia with use of HAS compared with alternatives (OR 0.524, 95% CI 0.395–0.696; *p*<0.0001). The use of HAS reduced the likelihood of developing hyponatremia in patients who underwent LVP OR 0.490 (95% CI 0.360–0.666; *p*<0.0001) (Figure 5B).

D. Serum sodium

A total of 37 studies reported the difference in serum sodium following HAS compared with alternatives. There was a rise in serum sodium in the HAS arm more than alternatives (WMD 1.474, 95% CI 0.645–2.304; *p*=0.0005) (Supplemental Materials, http://links.lww.com/HC9/C224).

E. HRS

HRS was reported in 10 studies. There was a 65.3% reduction in the overall odds of HRS with the use of HAS compared with alternative fluids (OR 0.347, 95% CI 0.145–0.831; *p*=0.0175). HAS was most beneficial in reducing the incidence of HRS in patients who underwent LVP, OR 0.245 (95% CI 0.062–0.971; *p*=0.0453) (Figure 5C).

F. PICD

PICD was reported in 15 studies. There is a 45.7% reduction in overall odds of PICD with use of HAS compared with alternatives (OR 0.543, 95% CI 0.351–0.840; *p*=0.006) (Figure 5D). Also, 26 studies reported plasma renin activity as a measure of PICD. There was a decrease in plasma renin activity following use of HAS compared with alternatives, with a pooled weighted mean difference of −1.568 (95% CI −2.606 to −0.529; *p*=0.003) (Supplemental Materials, http://links.lww.com/HC9/C224).

#### HE

Thirty-five studies reported HE with the use of HAS, leading to a non-significant 19.4% reduction in overall odds of HE (OR 0.806, 95% CI 0.619–1.048; *p*=0.1075) compared with alternative fluids. The overall pooled OR favored alternative fluids (Supplemental Materials, http://links.lww.com/HC9/C224).

#### Gastrointestinal bleeding and portal hypertensive bleeding

Gastrointestinal bleeding was reported in 28 studies with no differences between HAS and alternatives (OR 0.947, 95% CI 0.679–1.321; *p*=0.7497) (Supplemental Materials, http://links.lww.com/HC9/C224). Portal hypertensive bleeding was explicitly reported in 11 studies, with no difference between HAS and alternatives (OR 1.11, 95% CI 0.74–1.66; *p*=0.6229) (Supplemental Materials, http://links.lww.com/HC9/C224).

#### Recurrence of ascites

Ascites recurrence was reported in 26 studies, with a 30.6% reduction in the odds of ascites recurrence following HAS infusions compared with alternatives (OR 0.694, 95% CI 0.502–0.959; *p*=0.0267). HAS reduced recurrent ascites in patients treated with long-term HAS, OR 0.25 (95% CI 0.14–0.47; *p*<0.0001) (Figure 6).

**FIGURE 6:**
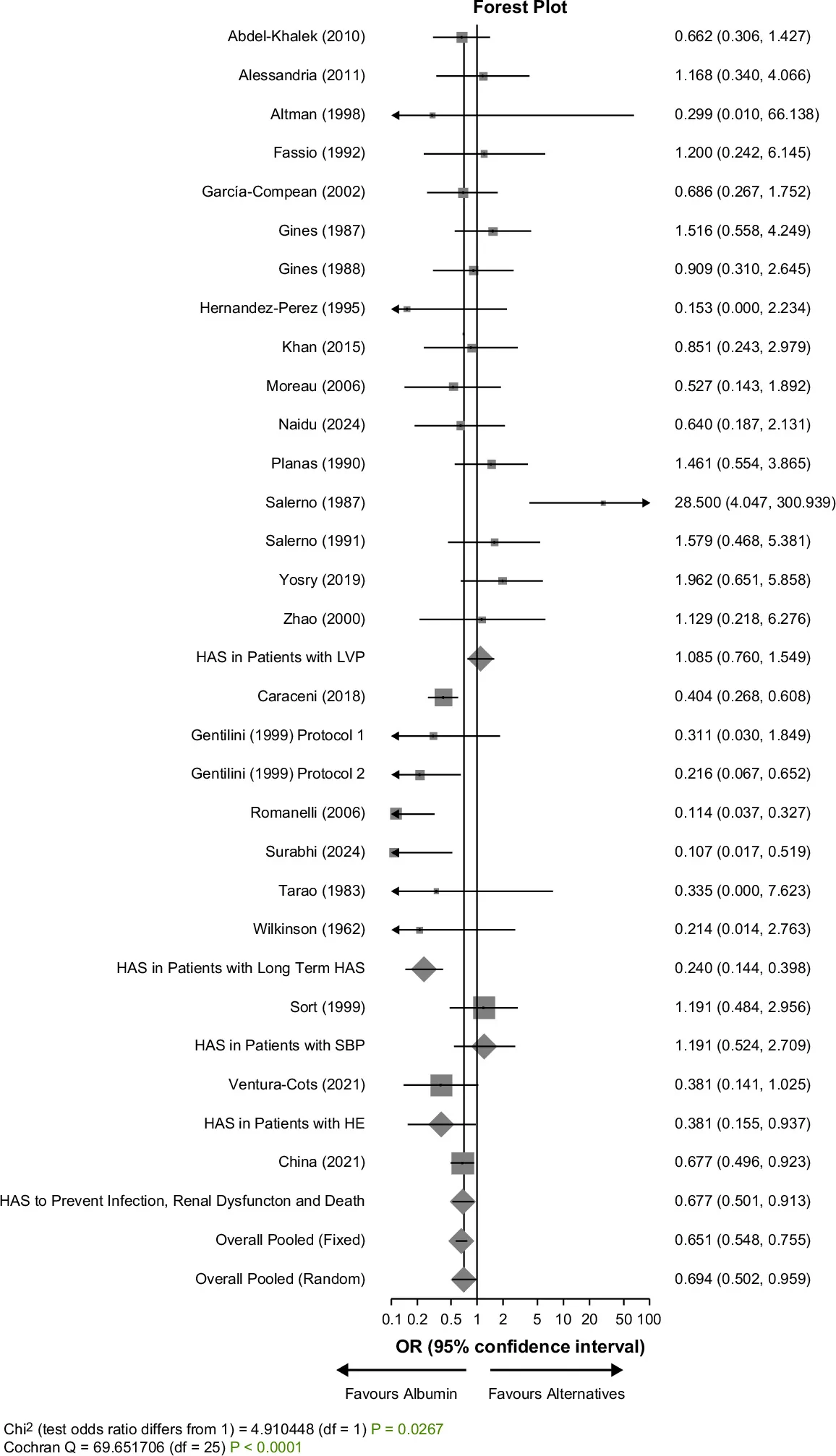
Recurrent ascites was reported in all trials (grouped according to clinical Indication for HAS use in liver cirrhosis) (OR, 95% CI). Abbreviations: HAS, human albumin solution; HE, hepatic encephalopathy; LVP, large volume paracentesis; SBP, spontaneous bacterial peritonitis.

#### Development of SBP and other bacterial Infections

- SBP

There were 19 studies that reported SBP as an outcome, with a non-significant 25.1% reduction in the odds of SBP (OR 0.749, 95% CI 0.472–1.190; *p*=0.2215) (Supplemental Materials, http://links.lww.com/HC9/C224).

B. Non-SBP (other bacterial infections, including sepsis)

There were 25 studies that reported other bacterial infections. Their overall random OR was not statistically significant (OR 0.887, 95% CI 0.647–1.215; *p*=0.4546) (Supplemental Materials, http://links.lww.com/HC9/C224).

#### Need for paracentesis

The need for paracentesis was reported in 6 studies. The studies' overall random OR of these studies was not statistically significant (OR 0.59, 95% CI 0.34–1.03; *p*=0.0656) (Supplemental Materials, http://links.lww.com/HC9/C224).

#### Multiorgan failure and ACLF

Multiorgan failure was reported in 5 studies. There was a non-significant reduction in the overall random odds ratio (OR 0.79, 95% CI 0.49–1.28; *p*=0.3481) (Supplemental Materials, http://links.lww.com/HC9/C224). ACLF was reported in only 3 studies (OR 0.66, 95% CI 0.37–1.18; *p*=0.1643) (Supplemental Materials, http://links.lww.com/HC9/C224).

## DISCUSSION

Our findings from 56 RCTs indicate that use of HAS is associated with an overall reduction in mortality and several cirrhosis-related complications, including renal impairment, hyponatremia, HRS, recurrent ascites, and PICD, consistent with previous meta-analyses.^6^^,^^7^^,^^9^ Bai et al.^7^ reported that HAS significantly reduced mortality, renal impairment, and ascites in cirrhotic patients, and Leache et al.^6^ showed HAS was associated with lower mortality among cirrhotic patients with SBP. We found no significant beneficial effect in reducing HE nor the development of bacterial infections, nor multiorgan failure. We also found no increase in gastrointestinal bleeding. These are consistent with other meta-analyses.^5^^,^^8^

However, most of the included trials were small and often of poor methodological quality. When trials were divided according to size, only the smaller-sized RCTs showed a significant mortality benefit following use of HAS in liver cirrhosis. This indicates that lower-quality trials accounted for the perceived overall mortality benefit, reducing our confidence in the results. Furthermore, 20 of the included trials in our qualitative risk of bias assessment were judged to have a high risk of bias. The dominance of small trials at high risk of bias limits the overall quality of the evidence and may have led to an overestimation of the treatment effect, thus distorting the true effect estimate in our meta-analysis.

Equally, our findings should be interpreted within the context of the healthcare setting of the included studies and the biological variability of HAS. Disparities in healthcare infrastructure and the availability of HAS in resource-limited inpatient settings due to constraints in institutional protocols may limit the generalizability of our findings. Also, biologic heterogeneity among cirrhotic patients according to disease severity or comorbidities may influence individual responses to HAS.

Our systematic review has several methodological limitations. First, there was substantial variability among the included trials. The studies differed in the doses of HAS infusion, the comparison groups, and the definitions of outcomes used. Although a random-effects estimator was employed to account for heterogeneity at the level of the trial, this variability likely contributed to different pooled effect estimates and wider confidence intervals. Second, there was methodological variability across the included trials. Differences in the study design and the trial quality may have influenced the observed treatment effect estimates and potentially overestimated the effectiveness of HAS. Publication bias also represents a challenge, particularly among smaller studies, which tend to receive greater weight in random-effects meta-analyses due to the common constant added to the variance of each study. This effect is evident when comparing random-effects results with the fixed-effects estimator, which produced a more modest (although still significant) estimate of the effect of HAS. Third, our analyses are based on combined published data rather than individual patient-level data, limiting our ability to perform individual patient-level analyses. In addition, a random-effects model and subgroup analyses were performed to address statistical heterogeneity. Yet heterogeneity remained present and remains a limitation of our findings. Lastly, the authors chose a cutoff point of 100 to distinguish between smaller, medium, and larger trials, based on the average sample size of the studies that reported mortality and renal impairment, as there is no universally accepted consensus for what constitutes a small, medium, or large sample size in the literature. Despite the number of studies in the larger-trials group being relatively small, potentially introducing bias, these findings remain important. There is often a bias against the publication of studies with negative or inconclusive results. However, the present data are informative for clinical practice and provide essential evidence to justify adequately powered trials, such as those funded through the Patient-Centered Outcomes Research Institute (PCORI).

Our review has a number of strengths. First, we conducted a comprehensive search strategy. Second, we reduced the risk of language bias by placing no language restrictions on our inclusion criteria. Third, full-text publications and abstracts were included to avoid time-lag bias. This approach ensured that recently conducted studies and abstracts presented at conferences were included.^13^ Lastly, this meta-analysis included more studies than previous meta-analyses, reducing the likelihood of publication bias by retrieving more evidence. Also, pooling data from multiple trials in this meta-analysis allowed us to obtain a more precise effect estimate of HAS. These strategies minimized the potential of reporting bias and increased the possibility of the overall pooled OR being close to the true effect of HAS.

In conclusion, HAS may be beneficial in reducing mortality and liver cirrhosis complications such as renal dysfunction and recurrent ascites. However, the predominance of small trials at high risk of bias and the presence of heterogeneity in this review suggest that these findings should be interpreted with caution. This is worrying given the importance of the clinical question, the widespread use of albumin, its expense, and the limitation of available treatment regimes. The available trials included 6714 patients and 1425 deaths altogether, with the largest trial only including 777 subjects. An RCT is urgently needed to provide more reliable evidence of clinically meaningful benefits. We advocate a large, well-designed, and adequately powered RCT to establish the true clinical effectiveness of HAS. We suggest a sample size of over 3000 evaluable liver cirrhosis patients, randomized 1:1 between albumin and control, with a 25% mortality rate, 90% power, and an alpha of 5% 2-sided would be required.

## Supplementary Material

## References

[1] Harmala S Parisinos C Ryan J O’Brien A . Effectiveness of intravenous albumin therapy to prevent spontaneous bacterial peritonitis, renal dysfunction and death in adults with cirrhosis: A protocol for a systematic review. BMJ Open. 2019;9:e025664. 10.1136/bmjopen-2018-025664 PMC6352793 30700489

[2] China L Freemantle N Forrest E Kallis Y Ryder SD Wright G . A randomized trial of albumin infusions in hospitalized patients with cirrhosis. N Engl J Med. 2021;384:808–817. 10.1056/NEJMoa2022166 33657293

[3] CDC—U.S. Centers for Disease Control and Prevention . FastStats—Chronic Liver Disease or Cirrhosis. Accessed July 9, 2025. https://www.cdc.gov/nchs/fastats/liver-disease.htm#print

[4] Office for Health Improvement & Disparities . Liver disease: Applying All Our Health. Accessed July 9, 2025. https://www.gov.uk/government/publications/liver-disease-applying-all-our-health/liver-disease-applying-all-our-health#fn:4

[5] Shrestha DB Budhathoki P Sedhai YR Baniya R Awal S Yadav J . Safety and efficacy of human serum albumin treatment in patients with cirrhotic ascites undergoing paracentesis: A systematic review and meta-analysis. Ann Hepatol. 2021;26:100547. 10.1016/j.aohep.2021.100547 34626828

[6] Leache L Gutiérrez-Valencia M Saiz LC Uriz J Bolado F García-Erce JA . Meta-analysis: Efficacy and safety of albumin in the prevention and treatment of complications in patients with cirrhosis. Aliment Pharmacol Ther. 2023;57:620–634. 10.1111/apt.17344 36524316

[7] Bai Z Wang L Wang R Zou M Méndez-Sánchez N Romeiro FG . Use of human albumin infusion in cirrhotic patients: A systematic review and meta-analysis of randomized controlled trials. Hepatol Int. 2022;16:1468–1483. 10.1007/s12072-022-10374-z 36048318

[8] Sandi BB Leao GS de Mattos AA de Mattos AZ . Long-term albumin administration in patients with cirrhosis and ascites: A meta-analysis of randomized controlled trials. J Gastroenterol Hepatol. 2021;36:609–617. 10.1111/jgh.15253 32914468

[9] Ashour AA Atta MA Sadek KW Obaid KR Ashour MA Ashour A . Albumin administration in patients with decompensated liver cirrhosis: A meta-analytic update. Eur J Gastroenterol Hepatol. 2021;33:479–486. 10.1097/MEG.0000000000001932 32976190

[10] Zaccherini G Tufoni M Bernardi M . Albumin administration is efficacious in the management of patients with cirrhosis: A systematic review of the literature. Hepat Med. 2020;12:153–172. 10.2147/HMER.S264231 PMC7602890 33149707

[11] Xu T Liu W Huang R . Can albumin reduce the mortality of patients with cirrhosis and ascites? A meta-analysis of randomized controlled trials. Eur J Gastroenterol Hepatol. 2023;35:80–88. 10.1097/MEG.0000000000002447 36165067

[12] Kar PS Venishetty S Laroia ST Jindal A Maiwall R Sood AK . Tolerance of standard dose albumin infused over 6 hrs for treatment of spontaneous bacterial peritonitis—A randomized controlled trial. Indian J Gastroenterol. 2023;42:505–516. 10.1007/s12664-023-01389-x 37422602

[13] O’Leary JG Perricone G Laleman W Milovanovic T Torres M Campins N . LBO-003 Efficacy and safety of long-term human albumin therapy in cirrhotic patients with acute decompensation and ascites: Topline results of the PRECIOSA trial. J Hepatol. 2025;82:S10.

[14] Sujith Reddy JSN Jagtap N Kalpala R Kulkarni A Gupta R Nagaraja Rao P . Midodrine versus albumin to prevent paracentesis induced circulatory dysfunction in acute on chronic liver failure patients in the outpatient clinic—A randomized controlled trial. J Clin Exp Hepatol. 2023;13:576–585. 10.1016/j.jceh.2023.01.009 PMC10333951 37440947

[15] Solà E Solé C Simón-Talero M Martín-Llahí M Castellote J Garcia-Martínez R . Midodrine and albumin for prevention of complications in patients with cirrhosis awaiting liver transplantation. A randomized placebo-controlled trial. J Hepatol. 2018;69:1250–1259. 10.1016/j.jhep.2018.08.006 30138685

