# Supplementary material for: A systematic review and meta-analysis of the use of human albumin solution for treating the complications of liver cirrhosis
Source: Hepatol Commun. 2026 Jan 16;10(2):e0875. doi: 10.1097/HC9.0000000000000875 (PMC13671596; doi:10.1097/HC9.0000000000000875)
Supplement: Supplementary file 1 [file hc9-10-e0875-s001.docx]

**SUPPLEMENTARY MATERIALS**

**TABLE OF CONTENTS:**

**1. PRISMA 2020 CHECKLIST	PAGE 2-7**

**2. SEARCH STRATEGY	PAGE 8-11**

**3. DATA EXTRACTION FORMS FOR BINARY OUTCOMES	PAGE 12-24**

I. **PRIMARY OUTCOME: MORTALITY**
II. **SECONDARY OUTCOMES:**
    a. **RENAL IMPAIRMENT**
    b. **HYPONATREMIA**
    c. **HEPATORENAL SYNDROME**
    d. **HEPATIC ENCEPHALOPATHY**
    e. **GASTROINTESTINAL BLEEDING**
    f. **PORTAL HYPERTENSIVE BLEEDING (INCLUDING VARICEAL BLEEDING)**
    g. **RECURRENCE OF ASCITES**
    h. **DEVELOPMENT OF BACTERIAL INFECTION**
       i. **SPONTANEOUS BACTERIAL PERITONITIS AND SEPSIS**
       ii. **NON- SPONTANEOUS BACTERIAL PERITONITIS**
    i. **PARACENTESIS INDUCED CIRCULATORY DYSFUNCTION**
    j. **NEED FOR PARACENTESIS**
    k. **MULTI-ORGAN FAILURE**
    l. **ACUTE-ON-CHRONIC- LIVER FAILURE**

**4. DATA EXTRACTION FORMS FOR CONTINIOUS OUTCOMES	PAGE 25-27**

I. **RENAL IMPAIRMENT**
   a. **SERUM CREATININE (MG/DL)**
   b. **SERUM SODIUM (MEQ/L)**
II. **CIRCULATORY DYSFUNCTION**
    a. **PLASMA RENIN ACTIVITY (NG/MG/HR)**

**5. REPORTING BIAS (FUNNEL PLOT)	PAGE 28**

**6. META-ANALYSIS OF BINARY OUTCOMES	PAGE 29-35**

**7. META-ANALYSIS OF CONTINIOUS OUTCOMES	PAGE 36-38**

**8. REFERENCES OF ALL RANDOMISED CONTROLELD TRIALS	PAGE 39-42**

# **1. PRISMA 2020 Main Checklist**

| **Topic** | **No.** | **Item** | **Location where item is reported** |
| --- | --- | --- | --- |
| **TITLE** |  |  |  |
| **Title** | 1 | Identify the report as a systematic review. | Page 1 |
| **ABSTRACT** |  |  |  |
| **Abstract** | 2 | See the PRISMA 2020 for Abstracts checklist | Page 2 |
| **INTRODUCTION** |  |  |  |
| **Rationale** | 3 | Describe the rationale for the review in the context of existing knowledge. | Page 3 |
| **Objectives** | 4 | Provide an explicit statement of the objective(s) or question(s) the review addresses. | Page 1 and 3 |
| **METHODS** |  |  |  |
| **Eligibility criteria** | 5 | Specify the inclusion and exclusion criteria for the review and how studies were grouped for the syntheses. | Page 4 |
| **Information sources** | 6 | Specify all databases, registers, websites, organisations, reference lists and other sources searched or consulted to identify studies. Specify the date when each source was last searched or consulted. | Page 4 |
| **Search strategy** | 7 | Present the full search strategies for all databases, registers and websites, including any filters and limits used. | supplementary materials |
| **Selection process** | 8 | Specify the methods used to decide whether a study met the inclusion criteria of the review, including how many reviewers screened each record and each report retrieved, whether they worked independently, and if applicable, details of automation tools used in the process. | Page 6 and Figure 1 |
| **Data collection process** | 9 | Specify the methods used to collect data from reports, including how many reviewers collected data from each report, whether they worked independently, any processes for obtaining or confirming data from study investigators, and if applicable, details of automation tools used in the process. | Page 6 and supplementary materials |
| **Data items** | 10a | List and define all outcomes for which data were sought. Specify whether all results that were compatible with each outcome domain in each study were sought (e.g. for all measures, time points, analyses), and if not, the methods used to decide which results to collect. | Page 5 and supplementary materials |
|  | 10b | List and define all other variables for which data were sought (e.g. participant and intervention characteristics, funding sources). Describe any assumptions made about any missing or unclear information. | Page 6 |
| **Study risk of bias assessment** | 11 | Specify the methods used to assess risk of bias in the included studies, including details of the tool(s) used, how many reviewers assessed each study and whether they worked independently, and if applicable, details of automation tools used in the process. | Page 6 and supplementary materials |
| **Effect measures** | 12 | Specify for each outcome the effect measure(s) (e.g. risk ratio, mean difference) used in the synthesis or presentation of results. | Page 6 |
| **Synthesis methods** | 13a | Describe the processes used to decide which studies were eligible for each synthesis (e.g. tabulating the study intervention characteristics and comparing against the planned groups for each synthesis (item 5)). | Page 6 and supplementary materials |
|  | 13b | Describe any methods required to prepare the data for presentation or synthesis, such as handling of missing summary statistics, or data conversions. | N/A |
|  | 13c | Describe any methods used to tabulate or visually display results of individual studies and syntheses. | page 6 and 7 |
|  | 13d | Describe any methods used to synthesize results and provide a rationale for the choice(s). If meta-analysis was performed, describe the model(s), method(s) to identify the presence and extent of statistical heterogeneity, and software package(s) used. | page 6 and 7 |
|  | 13e | Describe any methods used to explore possible causes of heterogeneity among study results (e.g. subgroup analysis, meta-regression). | page 7 |
|  | 13f | Describe any sensitivity analyses conducted to assess robustness of the synthesized results. | page 7 |
| **Reporting bias assessment** | 14 | Describe any methods used to assess risk of bias due to missing results in a synthesis (arising from reporting biases). | page 7 |
| **Certainty assessment** | 15 | Describe any methods used to assess certainty (or confidence) in the body of evidence for an outcome. | page 7 |
| **RESULTS** |  |  |  |
| **Study selection** | 16a | Describe the results of the search and selection process, from the number of records identified in the search to the number of studies included in the review, ideally using a flow diagram. | page 8 |
|  | 16b | Cite studies that might appear to meet the inclusion criteria, but which were excluded, and explain why they were excluded. | page 8 |
| **Study characteristics** | 17 | Cite each included study and present its characteristics. | table 1 (page 15 to 22) |
| **Risk of bias in studies** | 18 | Present assessments of risk of bias for each included study. | figure 2 (page 24 and 25) |
| **Results of individual studies** | 19 | For all outcomes, present, for each study: (a) summary statistics for each group (where appropriate) and (b) an effect estimate and its precision (e.g. confidence/credible interval), ideally using structured tables or plots. | table 2 (page 26) and figure 6 (page 33) |
| **Results of syntheses** | 20a | For each synthesis, briefly summarise the characteristics and risk of bias among contributing studies. | page 8 |
|  | 20b | Present results of all statistical syntheses conducted. If meta-analysis was done, present for each the summary estimate and its precision (e.g. confidence/credible interval) and measures of statistical heterogeneity. If comparing groups, describe the direction of the effect. | page 8 to 10 |
|  | 20c | Present results of all investigations of possible causes of heterogeneity among study results. | page 19 and supplementary materials |
|  | 20d | Present results of all sensitivity analyses conducted to assess the robustness of the synthesized results. | page 8 and 9 and Figure 3 and Figure 4 (page 27 and page 28) |
| **Reporting biases** | 21 | Present assessments of risk of bias due to missing results (arising from reporting biases) for each synthesis assessed. | supplementary materials |
| **Certainty of evidence** | 22 | Present assessments of certainty (or confidence) in the body of evidence for each outcome assessed. | page 8 to 10 and supplementary materials |
| **DISCUSSION** |  |  |  |
| **Discussion** | 23a | Provide a general interpretation of the results in the context of other evidence. | page 11 |
|  | 23b | Discuss any limitations of the evidence included in the review. | page 11 |
|  | 23c | Discuss any limitations of the review processes used. | page 11 and 12 |
|  | 23d | Discuss implications of the results for practice, policy, and future research. | page 12 |
| **OTHER INFORMATION** |  |  |  |
| **Registration and protocol** | 24a | Provide registration information for the review, including register name and registration number, or state that the review was not registered. | Page 4 |
|  | 24b | Indicate where the review protocol can be accessed, or state that a protocol was not prepared. | N/A |
|  | 24c | Describe and explain any amendments to information provided at registration or in the protocol. | N/A |
| **Support** | 25 | Describe sources of financial or non-financial support for the review, and the role of the funders or sponsors in the review. | Page 1 |
| **Competing interests** | 26 | Declare any competing interests of review authors. | Page 1 |
| **Availability of data, code and other materials** | 27 | Report which of the following are publicly available and where they can be found: template data collection forms; data extracted from included studies; data used for all analyses; analytic code; any other materials used in the review. | supplementary materials |

# PRISMA Abstract Checklist

| **Topic** | **No.** | **Item** | **Reported?** |
| --- | --- | --- | --- |
| **TITLE** |  |  |  |
| **Title** | 1 | Identify the report as a systematic review. | Yes |
| **BACKGROUND** |  |  |  |
| **Objectives** | 2 | Provide an explicit statement of the main objective(s) or question(s) the review addresses. | Yes |
| **METHODS** |  |  |  |
| **Eligibility criteria** | 3 | Specify the inclusion and exclusion criteria for the review. | Yes |
| **Information sources** | 4 | Specify the information sources (e.g. databases, registers) used to identify studies and the date when each was last searched. | Yes |
| **Risk of bias** | 5 | Specify the methods used to assess risk of bias in the included studies. | No |
| **Synthesis of results** | 6 | Specify the methods used to present and synthesize results. | No |
| **RESULTS** |  |  |  |
| **Included studies** | 7 | Give the total number of included studies and participants and summarise relevant characteristics of studies. | Yes |
| **Synthesis of results** | 8 | Present results for main outcomes, preferably indicating the number of included studies and participants for each. If meta-analysis was done, report the summary estimate and confidence/credible interval. If comparing groups, indicate the direction of the effect (i.e. which group is favoured). | Yes |
| **DISCUSSION** |  |  |  |
| **Limitations of evidence** | 9 | Provide a brief summary of the limitations of the evidence included in the review (e.g. study risk of bias, inconsistency and imprecision). | Yes |
| **Interpretation** | 10 | Provide a general interpretation of the results and important implications. | Yes |
| **OTHER** |  |  |  |
| **Funding** | 11 | Specify the primary source of funding for the review. | No |
| **Registration** | 12 | Provide the register name and registration number. | No |

*From:* Page MJ, McKenzie JE, Bossuyt PM, Boutron I, Hoffmann TC, Mulrow CD, et al. The PRISMA 2020 statement: an updated guideline for reporting systematic reviews. MetaArXiv. 2020, September 14. DOI: 10.31222/osf.io/v7gm2. For more information, visit: www.prisma-statement.org

**2. SEARCH STRATEGY:**

**Search “concepts” are:**

**“LIVER CIRRHOSIS”, “ALBUMIN”, “RANDOMIZED CONTROL TRIALS”**

**Databases:**

**(1) Cochrane Library (CENTRAL) <1947 to 2025 April 02>**

**(2) Ovid MEDLINE(R) ALL <1946 to April 02, 2025>**

**(3) Embase Classic+Embase <1947 to 2025 April 02>**

(1) **DATABASE : CENTRAL** 🡪 **PLATFORM; COCHRANE LIBRARY**

| # | **Query** | **Results from 2 April 2025** |
| --- | --- | --- |
| #1 | MeSH descriptor: [Liver Cirrhosis] explode all trees | 4078 |
| #2 | (Cirrho*):ti,ab,kw | 13381 |
| #3 | #1 or #2 | 13381 |
| #4 | MeSH descriptor: [Serum Albumin] this term only | 1378 |
| #5 | MeSH descriptor: [Serum Albumin, Human] this term only | 123 |
| #6 | MeSH descriptor: [Serum Albumin, Radio-Iodinated] this term only | 20 |
| #7 | (albumin*):ti,ab,kw | 21529 |
| #8 | #4 or #5 or #6 or #7 | 21529 |
| #9 | #3 AND #8 in Trials | **1235** |

| # | Searches | Results on  April 02 2025 |
| --- | --- | --- |
| 1 | exp Liver Cirrhosis/ | 108354 |
| 2 | Cirrho*.ti,ab,kw. | 125136 |
| 3 | 1 or 2 | 165872 |
| 4 | serum albumin/ or serum albumin, human/ or serum albumin, radio-iodinated/ | 57256 |
| 5 | albumin*.ti,ab,kw. | 199270 |
| 6 | 4 or 5 | 218542 |
| 7 | randomized controlled trial.pt. | 635556 |
| 8 | controlled clinical trial.pt. | 95689 |
| 9 | randomized.ab. | 686179 |
| 10 | placebo.ab. | 257326 |
| 11 | drug therapy.fs. | 2799654 |
| 12 | randomly.ab. | 456845 |
| 13 | trial.ab. | 746401 |
| 14 | groups.ab. | 2832614 |
| 15 | 7 or 8 or 9 or 10 or 11 or 12 or 13 or 14 | 6252610 |
| 16 | 3 and 6 and 15 | 2528 |
| 17 | exp animals/ not humans.sh. | 5326238 |
| 18 | 16 not 17 | **2251** |

(2) **DATABASE: Ovid MEDLINE** 🡪 **PLATFORM; OVIDSP**
(3) **DATABASE : EMBASE (Embase classic +Embase)** 🡪 **PLATFORM; OVIDSP**

| # | Searches | Results on  April 02 2025 |
| --- | --- | --- |
| 1 | Cirrho*.ti,ab,kw. | 198754 |
| 2 | exp liver cirrhosis/ | 207845 |
| 3 | 1 or 2 | 255598 |
| 4 | albumin/ | 189511 |
| 5 | serum albumin/ | 43301 |
| 6 | radioiodinated human serum albumin/ | 748 |
| 7 | albumin*.ti,ab,kw. | 276715 |
| 8 | 4 or 5 or 6 or 7 | 355570 |
| 9 | Randomized controlled trial/ | 875069 |
| 10 | Controlled clinical study/ | 445272 |
| 11 | Random$.ti,ab. | 2190537 |
| 12 | randomization/ | 100555 |
| 13 | intermethod comparison/ | 313483 |
| 14 | placebo.ti,ab. | 391019 |
| 15 | (compare or compared or comparison).ti. | 648033 |
| 16 | ((evaluated or evaluate or evaluating or assessed or assess) and (compare or compared or comparing or comparison)).ab. | 3112140 |
| 17 | (open adj label).ti,ab. | 123026 |
| 18 | ((double or single or doubly or singly) adj (blind or blinded or blindly)).ti,ab. | 292996 |
| 19 | double blind procedure/ | 230579 |
| 20 | parallel group$1.ti,ab. | 35257 |
| 21 | (crossover or cross over).ti,ab. | 133276 |
| 22 | ((assign$ or match or matched or allocation) adj5 (alternate or group$1 or intervention$1 or patient$1 or subject$1 or participant$1)).ti,ab. | 456081 |
| 23 | (assigned or allocated).ti,ab. | 539492 |
| 24 | (controlled adj7 (study or design or trial)).ti,ab. | 500108 |
| 25 | (volunteer or volunteers).ti,ab. | 297072 |
| 26 | trial.ti. | 453514 |
| 27 | or/9-26 | 6459829 |
| 28 | 3 and 8 and 27 | 5587 |
| 29 | (exp animal/ or animal.hw. or nonhuman/) not (exp human/ or human cell/ or (human or humans).ti.) | 7635737 |
| 30 | 28 not 29 | **5299** |

**3. DATA EXTRACTION FORMS FOR BINARY OUTCOMES:**

Mortality:

| Study Name | Albumin Events | Albumin Total | Control Events | Control Total |
| --- | --- | --- | --- | --- |
| Abdul-Khalek (2010) | 5 | 68 | 6 | 67 |
| Alessandria (2011) | 3 | 35 | 3 | 35 |
| Appenrodt (2008) | 0 | 13 | 0 | 11 |
| Arora (2020) | 11 | 40 | 25 | 40 |
| Bari (2012) | 4 | 13 | 5 | 12 |
| Bertran (1991) | 1 | 8 | 0 | 9 |
| Bruno (1992) | 1 | 18 | 0 | 17 |
| Caraceni (2018) | 38 | 218 | 46 | 213 |
| Chen (2009) | 4 | 15 | 6 | 15 |
| China (2021) | 132 | 380 | 119 | 397 |
| Degoricija (2003) | 0 | 10 | 0 | 40 |
| Devisetty (2024) | 23 | 116 | 14 | 58 |
| Fassio (1992) | 6 | 21 | 6 | 20 |
| Fernandez (2020) | 17 | 61 | 13 | 57 |
| García-Compean (1993) | 0 | 17 | 0 | 18 |
| García-Compean (2002) | 11 | 48 | 18 | 48 |
| Gentilini (1999) Protocol 1 | 1 | 63 | 0 | 63 |
| Gentilini (1999) Protocol 2 | 11 | 43 | 9 | 38 |
| Gines (1987) | 23 | 58 | 16 | 59 |
| Gines (1988) | 18 | 52 | 14 | 53 |
| Gines (1996) | 2 | 97 | 10 | 192 |
| Guevara (2012) | 8 | 46 | 10 | 51 |
| Hamdy (2014) | 0 | 25 | 7 | 25 |
| Hernandez-Perez (1995) | 0 | 8 | 0 | 8 |
| Jalan (2007) | 1 | 12 | 5 | 12 |
| Kang (1998) | 0 | 6 | 0 | 6 |
| Kar (2023) | 3 | 31 | 5 | 32 |
| Khan (2015) | 0 | 25 | 0 | 25 |
| Luca (1995) | 0 | 9 | 0 | 9 |
| Maiwall (2022) | 29 | 50 | 31 | 50 |
| Maiwall (2023) | 2 | 52 | 8 | 54 |
| Maiwall (2024) | 5 | 61 | 15 | 60 |
| Moreau (2002) | 1 | 10 | 1 | 10 |
| Moreau (2006) | 1 | 30 | 3 | 38 |
| O'Leary (2025) | 32 | 203 | 41 | 207 |
| Philips (2021) | 87 | 154 | 95 | 154 |
| Planas (1990) | 13 | 43 | 17 | 45 |
| Reddy (2023) | 3 | 25 | 4 | 25 |
| Romanelli (2006) | 29 | 54 | 25 | 46 |
| Salerno (1987) | 3 | 20 | 4 | 21 |
| Salerno (1991) | 13 | 27 | 14 | 27 |
| Salman (2016) | 22 | 100 | 28 | 100 |
| Sharma (2017) | 11 | 60 | 19 | 60 |
| Simon-Talero (2013) | 6 | 26 | 14 | 30 |
| Singh (2008) | 0 | 20 | 1 | 20 |
| Sola (2018) | 6 | 87 | 4 | 86 |
| Sola Vera (2003) | 1 | 37 | 1 | 35 |
| Sort (1999) | 14 | 63 | 26 | 63 |
| Tarao (1983) | 0 | 7 | 3 | 10 |
| Thevenot (2015) | 27 | 96 | 20 | 95 |
| Ventura-Cots (2021) | 5 | 40 | 11 | 41 |
| Wilkinson (1962) | 3 | 7 | 3 | 9 |
| Xue (2002) | 5 | 56 | 17 | 56 |
| Yosry (2019) | 2 | 25 | 2 | 50 |
| Zaak (2001) | 8 | 21 | 5 | 14 |
| Zhao (2000) | 14 | 36 | 11 | 32 |

Renal Impairment:

| Study Name | Albumin Events | Albumin Total | Control Events | Control Total |
| --- | --- | --- | --- | --- |
| Abdul-Khalek (2010) | 1 | 68 | 1 | 67 |
| Alessandria (2011) | 0 | 35 | 0 | 35 |
| Altman (1998) | 0 | 33 | 0 | 27 |
| Appenrodt (2008) | 0 | 13 | 2 | 11 |
| Arora (2020) | 12 | 40 | 25 | 40 |
| Bari (2012) | 0 | 13 | 1 | 12 |
| Bertan (1991) | 1 | 8 | 0 | 9 |
| Caraceni (2018) | 1 | 218 | 2 | 213 |
| Chen (2009) | 1 | 15 | 3 | 15 |
| China (2021) | 40 | 380 | 57 | 397 |
| Fassio (1992) | 1 | 21 | 1 | 20 |
| Fernandez (2005) | 0 | 10 | 1 | 10 |
| Fernandez (2020) | 10 | 61 | 5 | 57 |
| García-Compean (1993) | 1 | 17 | 2 | 18 |
| García-Compean (2002) | 7 | 48 | 2 | 48 |
| Gentilini (1999) Protocol 1 | 0 | 63 | 2 | 63 |
| Gentilini (1999) Protocol 2 | 4 | 43 | 2 | 38 |
| Gines (1987) | 2 | 58 | 16 | 59 |
| Gines (1988) | 0 | 52 | 6 | 53 |
| Gines (1996) | 7 | 97 | 18 | 192 |
| Guevara (2012) | 1 | 46 | 3 | 51 |
| Hamdy (2014) | 0 | 25 | 7 | 25 |
| Hernandez-Perez (1995) | 1 | 8 | 0 | 8 |
| Jalan (2007) | 1 | 12 | 5 | 12 |
| Jha (2023) | 0 | 25 | 0 | 25 |
| Kang (1998) | 0 | 6 | 0 | 6 |
| Kar (2023) | 3 | 31 | 4 | 32 |
| Maiwall (2022) | 24 | 50 | 31 | 50 |
| Maiwall (2023) | 6 | 52 | 14 | 54 |
| Maiwall (2024) | 8 | 61 | 26 | 60 |
| Moreau (2002) | 0 | 10 | 0 | 10 |
| Moreau (2006) | 4 | 30 | 8 | 38 |
| Naidu (2024) | 6 | 33 | 8 | 33 |
| Planas (1990) | 0 | 43 | 0 | 45 |
| Reddy (2023) | 3 | 25 | 1 | 25 |
| Salerno (1987) | 1 | 20 | 1 | 21 |
| Salerno (1991) | 1 | 27 | 1 | 27 |
| Salman (2016) | 14 | 100 | 22 | 100 |
| Singh (2006) Noradrenaline | 0 | 20 | 0 | 20 |
| Singh (2006) Terlipressin | 1 | 20 | 1 | 20 |
| Singh (2008) | 0 | 20 | 0 | 20 |
| Sola Vera (2003) | 2 | 37 | 3 | 35 |
| Sola (2018) | 12 | 87 | 11 | 86 |
| Sort (1999) | 6 | 63 | 21 | 63 |
| Thevenot (2015) | 13 | 91 | 12 | 89 |
| Xue (2002) | 5 | 56 | 19 | 56 |
| Yosry (2019) | 3 | 25 | 7 | 50 |
| Zaak (2001) | 3 | 21 | 1 | 14 |

Hyponatremia:

| Study Name | Albumin Events | Albumin Total | Control Events | Control Total |
| --- | --- | --- | --- | --- |
| Abdul-Khalek (2010) | 2 | 68 | 3 | 67 |
| Alessandria (2011) | 2 | 35 | 3 | 35 |
| Altman (1998) | 0 | 33 | 1 | 27 |
| Appenrodt (2008) | 7 | 13 | 4 | 11 |
| Arora (2020) | 9 | 40 | 27 | 40 |
| Bertan (1991) | 0 | 8 | 2 | 9 |
| Bruno (1992) | 1 | 18 | 1 | 17 |
| Fassio (1992) | 4 | 21 | 3 | 20 |
| García-Compean (1993) | 1 | 17 | 1 | 18 |
| García-Compean (2002) | 3 | 48 | 5 | 48 |
| Gentilini (1999) Protocol 1 | 1 | 63 | 5 | 63 |
| Gines (1987) | 3 | 58 | 18 | 59 |
| Gines (1988) | 1 | 52 | 9 | 53 |
| Gines (1996) | 14 | 97 | 42 | 192 |
| Hernandez-Perez (1995) | 1 | 8 | 1 | 8 |
| Kang (1998) | 0 | 6 | 0 | 6 |
| Moreau (2002) | 1 | 10 | 1 | 10 |
| Moreau (2006) | 5 | 30 | 11 | 38 |
| Naidu (2024) | 9 | 33 | 11 | 33 |
| Planas (1990) | 3 | 43 | 4 | 45 |
| Reddy (2023) | 0 | 25 | 1 | 25 |
| Salerno (1991) | 4 | 27 | 5 | 27 |
| Singh (2006) Noradrenaline | 0 | 20 | 0 | 20 |
| Singh (2006) Terlipressin | 0 | 20 | 2 | 20 |
| Singh (2008) | 0 | 20 | 0 | 20 |
| Sola Vera (2003) | 2 | 37 | 5 | 35 |
| Sola (2018) | 11 | 87 | 14 | 86 |
| Ventura-Cots (2021) | 4 | 40 | 2 | 41 |
| Yosry (2019) | 4 | 25 | 11 | 50 |
| Zaak (2001) | 3 | 21 | 4 | 14 |

Hepatorenal Syndrome:

| Study Name | Albumin Events | Albumin Total | Control Events | Control Total |
| --- | --- | --- | --- | --- |
| Appenrodt (2008) | 0 | 13 | 1 | 11 |
| Bari (2012) | 0 | 13 | 0 | 12 |
| Caraceni (2018) | 1 | 218 | 7 | 213 |
| Fassio (1992) | 1 | 21 | 1 | 20 |
| Fernandez (2005) | 0 | 10 | 1 | 10 |
| García-Compean (2002) | 1 | 48 | 3 | 48 |
| Guevara (2012) | 1 | 56 | 4 | 54 |
| Hamdy (2014) | 0 | 25 | 9 | 25 |
| Romanelli (2006) | 1 | 54 | 0 | 46 |
| Sharma (2017) | 2 | 60 | 1 | 60 |

Paracentesis Induced Circulatory Dysfunction (PICD):

| Study Name | Albumin Events | Albumin Total | Control Events | Control Total |
| --- | --- | --- | --- | --- |
| Abdul-Khalek (2010) | 6 | 68 | 16 | 67 |
| Alessandria (2011) | 7 | 35 | 5 | 35 |
| AlSebaey (2013) | 6 | 50 | 9 | 75 |
| Appenrodt (2008) | 4 | 13 | 6 | 11 |
| Arora (2020) | 12 | 40 | 28 | 40 |
| Bari (2012) | 2 | 11 | 2 | 8 |
| China (2021) | 26 | 380 | 20 | 397 |
| Fernandez (2005) | 0 | 10 | 3 | 10 |
| García-Compean (2002) | 10 | 48 | 20 | 48 |
| Gines (1996) | 17 | 92 | 68 | 188 |
| Reddy (2023) | 4 | 25 | 5 | 25 |
| Singh (2006) Noradrenaline | 1 | 20 | 2 | 20 |
| Singh (2006) Terlipressin | 2 | 20 | 2 | 20 |
| Singh (2008) | 2 | 20 | 0 | 20 |
| Sola Vera (2003) | 4 | 37 | 11 | 35 |

Hepatic Encephalopathy:

| Study Name | Albumin Events | Albumin Total | Control Events | Control Total |
| --- | --- | --- | --- | --- |
| Abdul-Khalek (2010) | 3 | 68 | 2 | 67 |
| Alessandria (2011) | 4 | 35 | 4 | 35 |
| Altman (1998) | 1 | 33 | 0 | 27 |
| Arora (2020) | 11 | 40 | 20 | 40 |
| Baik (2000) | 0 | 11 | 0 | 12 |
| Bari (2012) | 1 | 13 | 0 | 12 |
| Bertan (1991) | 1 | 8 | 0 | 9 |
| China (2021) | 4 | 380 | 1 | 397 |
| Fagan (2023) | 17 | 24 | 23 | 24 |
| Fassio (1992) | 1 | 21 | 2 | 20 |
| García-Compean (1993) | 1 | 17 | 0 | 18 |
| Gentilini (1999) Protocol 1 | 2 | 63 | 2 | 63 |
| Gentilini (1999) Protocol 2 | 7 | 43 | 2 | 38 |
| Gines (1987) | 14 | 54 | 14 | 46 |
| Gines (1988) | 6 | 52 | 3 | 53 |
| Gines (1996) | 3 | 97 | 10 | 192 |
| Hamdy (2014) | 0 | 25 | 7 | 25 |
| Jalan (2007) | 1 | 12 | 5 | 12 |
| Kang (1998) | 0 | 6 | 0 | 6 |
| Lata (2007) | 1 | 25 | 0 | 24 |
| Maiwall (2023) | 3 | 52 | 5 | 54 |
| Maiwall (2024) | 2 | 61 | 9 | 60 |
| Moreau (2006) | 7 | 30 | 9 | 38 |
| Planas (1990) | 3 | 43 | 3 | 45 |
| Reddy (2023) | 3 | 25 | 2 | 25 |
| Romanelli (2006) | 11 | 54 | 11 | 46 |
| Salerno (1987) | 2 | 20 | 3 | 21 |
| Salerno (1991) | 2 | 27 | 2 | 27 |
| Sharma (2017) | 15 | 60 | 28 | 60 |
| Simon-Talero (2013) | 15 | 26 | 20 | 30 |
| Sola (2018) | 24 | 87 | 21 | 86 |
| Ventura-Cots (2021) | 11 | 40 | 8 | 41 |
| Yosry (2019) | 1 | 25 | 1 | 50 |
| Zaak (2001) | 3 | 21 | 3 | 14 |
| Zhao (2000) | 2 | 36 | 2 | 32 |

Gastrointestinal Bleeding:

| Study Name | Albumin Events | Albumin Total | Control Events | Control Total |
| --- | --- | --- | --- | --- |
| Abdul-Khalek (2010) | 5 | 68 | 5 | 67 |
| Alessandria (2011) | 0 | 35 | 0 | 35 |
| Altman (1998) | 0 | 33 | 1 | 27 |
| Bari (2012) | 1 | 13 | 0 | 12 |
| Caraceni (2018) | 2 | 218 | 1 | 213 |
| China (2021) | 11 | 380 | 13 | 397 |
| Fassio (1992) | 3 | 21 | 0 | 20 |
| Fernandez (2020) | 4 | 61 | 3 | 57 |
| García-Compean (2002) | 4 | 48 | 7 | 48 |
| Gentilini (1999) Protocol 2 | 3 | 43 | 1 | 38 |
| Gines (1987) | 2 | 58 | 6 | 59 |
| Gines (1988) | 2 | 52 | 1 | 53 |
| Gines (1996) | 1 | 97 | 5 | 192 |
| Hamdy (2014) | 0 | 25 | 1 | 25 |
| Planas (1990) | 3 | 41 | 7 | 43 |
| Reddy (2023) | 0 | 25 | 1 | 25 |
| Salerno (1987) | 0 | 20 | 2 | 21 |
| Salerno (1991) | 3 | 27 | 0 | 27 |
| Sharma (2017) | 15 | 60 | 13 | 60 |
| Simon-Talero (2013) | 1 | 26 | 3 | 30 |
| Singh (2008) | 0 | 20 | 1 | 20 |
| Sola Vera (2003) | 1 | 37 | 0 | 35 |
| Sola (2018) | 8 | 87 | 4 | 86 |
| Thevenot (2015) | 3 | 96 | 4 | 97 |
| Ventura-Cots (2021) | 2 | 40 | 0 | 41 |
| Xue (2002) | 1 | 56 | 2 | 56 |
| Zaak (2001) | 1 | 21 | 2 | 14 |
| Zhao (2000) | 5 | 36 | 2 | 32 |

Portal Hypertensive Bleeding:

| Study Name | Albumin Events | Albumin Total | Control Events | Control Total |
| --- | --- | --- | --- | --- |
| Alessandria (2011) | 0 | 35 | 1 | 35 |
| Caraceni (2018) | 5 | 38 | 2 | 46 |
| China (2021) | 31 | 380 | 32 | 397 |
| Fernandez (2020) | 4 | 61 | 2 | 57 |
| Gentilini (1999) Protocol 1 | 1 | 63 | 0 | 63 |
| Moreau (2006) | 4 | 30 | 3 | 38 |
| Planas (1990) | 1 | 43 | 0 | 45 |
| Romanelli (2006) | 5 | 54 | 10 | 46 |
| Sola-Vera (2003) | 1 | 37 | 0 | 35 |
| Thevenot (2015) | 1 | 95 | 0 | 96 |
| Wilkinson (1962) | 1 | 7 | 0 | 9 |

Recurrence of Ascites:

| Study Name | Albumin Events | Albumin Total | Control Events | Control Total |
| --- | --- | --- | --- | --- |
| Abdel-Khalek (2010) | 21 | 68 | 27 | 67 |
| Alessandria (2011) | 9 | 35 | 8 | 35 |
| Altman (1998) | 0 | 33 | 1 | 27 |
| Caraceni (2018) | 71 | 218 | 116 | 213 |
| China (2021) | 236 | 380 | 281 | 397 |
| Fassio (1992) | 6 | 21 | 5 | 20 |
| García-Compean (2002) | 30 | 48 | 34 | 48 |
| Gentilini (1999) Protocol 1 | 2 | 63 | 6 | 63 |
| Gentilini (1999) Protocol 2 | 21 | 43 | 31 | 38 |
| Gines (1987) | 16 | 54 | 10 | 46 |
| Gines (1988) | 10 | 52 | 11 | 53 |
| Hernandez-Perez (1995) | 0 | 8 | 2 | 8 |
| Khan (2015) | 11 | 25 | 12 | 25 |
| Moreau (2006) | 21 | 30 | 31 | 38 |
| Naidu (2024) | 8 | 33 | 11 | 33 |
| Planas (1990) | 18 | 41 | 15 | 43 |
| Romanelli (2006) | 21 | 54 | 39 | 46 |
| Salerno (1987) | 15 | 20 | 2 | 21 |
| Salerno (1991) | 13 | 27 | 10 | 27 |
| Sort (1999) | 16 | 63 | 14 | 63 |
| Surabhi (2024) | 11 | 25 | 22 | 25 |
| Tarao (1983) | 0 | 7 | 1 | 10 |
| Ventura-Cots (2021) | 14 | 40 | 24 | 41 |
| Wilkinson (1962) | 3 | 7 | 7 | 9 |
| Yosry (2018) | 12 | 25 | 16 | 50 |
| Zhao (2000) | 5 | 36 | 4 | 32 |

Development of Bacterial Infection

Spontaneous Bacterial Peritonitis (SBP):

| Study Name (SBP) | Albumin | Total | Comparison | Total |
| --- | --- | --- | --- | --- |
| Alessandria (2011) | 1 | 35 | 0 | 35 |
| China (2021) | 0 | 380 | 5 | 397 |
| Fernandez (2020) | 1 | 61 | 0 | 57 |
| Gentilini (1999) Protocol 2 | 2 | 43 | 3 | 38 |
| Gines (1987) | 0 | 58 | 4 | 59 |
| Gines (1988) | 0 | 52 | 2 | 53 |
| Kar (2023) | 2 | 31 | 0 | 32 |
| Moreau (2006) | 2 | 30 | 4 | 38 |
| Planas (1990) | 0 | 43 | 2 | 45 |
| Romanelli (2006) | 3 | 54 | 2 | 46 |
| Salerno (1991) | 1 | 27 | 0 | 27 |
| Salman (2016) | 5 | 100 | 6 | 100 |
| Sharma (2017) | 13 | 60 | 14 | 60 |
| Singh (2008) | 1 | 20 | 2 | 20 |
| Sola-Vera (2003) | 1 | 37 | 1 | 35 |
| Sort (1999) | 1 | 63 | 4 | 63 |
| Ventura-Cots (2021) | 1 | 40 | 1 | 41 |
| Zaak (2001) | 0 | 21 | 1 | 14 |
| Zhao (2000) | 2 | 36 | 1 | 32 |

Non-Spontaneous Bacterial Peritonitis (Non-SBP):

| Study Name (Non-SBP Bacterial Infections) | Albumin | Total | Comparison | Total |
| --- | --- | --- | --- | --- |
| Abdel-Khalek (2010) | 3 | 68 | 3 | 67 |
| Alessandria (2011) | 0 | 35 | 1 | 35 |
| Altman (1998) | 2 | 33 | 3 | 27 |
| Arora (2020) | 9 | 40 | 19 | 40 |
| Caraceni (2018) | 13 | 38 | 12 | 46 |
| China (2021) | 79 | 380 | 71 | 397 |
| Fagan (2023) | 0 | 24 | 3 | 24 |
| Fassio (1992) | 1 | 21 | 2 | 20 |
| Fernandez (2020) | 10 | 61 | 20 | 57 |
| García-Compean (2002) | 2 | 48 | 4 | 48 |
| Gines (1987) | 5 | 54 | 5 | 46 |
| Gines (1988) | 4 | 52 | 2 | 53 |
| Gines (1996) | 5 | 97 | 6 | 192 |
| Jalan (2007) | 3 | 12 | 7 | 12 |
| Maiwall (2023) | 13 | 52 | 20 | 54 |
| Moreau (2006) | 1 | 30 | 6 | 38 |
| Planas (1990) | 2 | 43 | 2 | 45 |
| Reddy (2023) | 3 | 25 | 3 | 25 |
| Romanelli (2006) | 2 | 54 | 0 | 46 |
| Salerno (1991) | 1 | 27 | 0 | 27 |
| Sharma (2017) | 14 | 60 | 6 | 60 |
| Sola (2018) | 12 | 87 | 13 | 86 |
| Sola-Vera (2003) | 2 | 37 | 1 | 35 |
| Sort (1999) | 0 | 63 | 2 | 63 |
| Ventura-Cots (2021) | 21 | 40 | 13 | 41 |

Need for Paracentesis:

| Study Name | Albumin Events | Albumin Total | Control Events | Control Total |
| --- | --- | --- | --- | --- |
| Altman (1998) | 6 | 33 | 11 | 27 |
| Bari (2012) | 5 | 13 | 4 | 12 |
| Caraceni (2018) | 18 | 218 | 42 | 213 |
| Singh (2006) Terlipressin | 2 | 20 | 1 | 20 |
| Singh (2008) | 2 | 20 | 1 | 20 |
| Sort (1999) | 14 | 63 | 16 | 63 |

Multi-Organ Failure:

| Study Name | Albumin Events | Albumin Total | Control Events | Control Total |
| --- | --- | --- | --- | --- |
| China (2021) | 23 | 380 | 31 | 397 |
| Reddy (2023) | 3 | 25 | 3 | 25 |
| Simon-Talero (2013) | 2 | 26 | 7 | 30 |
| Sola (2018) | 4 | 87 | 2 | 86 |
| Thevenot (2015) | 1 | 91 | 0 | 89 |

Acute-on-Chronic Liver Failure:

| Study Name | Albumin Events | Albumin Total | Control Events | Control Total |
| --- | --- | --- | --- | --- |
| Caraceni (2018) | 8 | 38 | 15 | 46 |
| Fernandez (2020) | 12 | 61 | 13 | 57 |
| Romanelli (2006) | 5 | 54 | 7 | 46 |

**4. DATA EXTRACTION FORMS FOR CONTINIOUS OUTCOMES:**

Renal Dysfunction - Serum Creatinine:

| Study Name | Serum Creatinine (In Albumin Group) | SD | Total Albumin Group Participants | Serum Creatinine (In Control Group) | SD | Total Control Group Participants |
| --- | --- | --- | --- | --- | --- | --- |
| Abdul-Khalek (2010) | 1.3 | 0.08 | 68 | 1.3 | 0.08 | 67 |
| Alessandria (2011) | 0.89 | 0.2 | 35 | 0.79 | 0.4 | 35 |
| Altman (1998) | 0.91 | 0.29 | 33 | 0.91 | 0.25 | 27 |
| Appenrodt (2008) | 1 | 0.17 | 13 | 0.97 | 0.2 | 11 |
| Arora (2020) | 1.13 | 0.44 | 40 | 1.89 | 0.58 | 40 |
| Baik (2000) | 0.82 | 0.17 | 11 | 0.96 | 0.29 | 12 |
| Bari (2012) | 0.93 | 0.39 | 11 | 1.27 | 1.02 | 9 |
| Chen (2009) | 1.2 | 1 | 15 | 1.1 | 0.4 | 15 |
| Degoricija (2003) | 1.13 | 0.441 | 10 | 0.868 | 0.303 | 40 |
| Devisetty (2024) | 0.82 | 0.378 | 104 | 0.86 | 0.511 | 54 |
| Fassio (1992) | 1.1 | 0.2 | 21 | 1 | 0.2 | 20 |
| Fernandez (2005) | 1 | 0.3 | 10 | 1 | 0.2 | 10 |
| Fernandez (2020) | 1.1 | 0.6 | 61 | 1.1 | 0.4 | 57 |
| García-Compean (1993) | 1.06 | 0.72 | 17 | 1.09 | 0.6 | 18 |
| García-Compean (2002) | 0.8 | 0.4 | 48 | 0.9 | 0.3 | 48 |
| Gines (1987) | 1.1 | 0.1 | 58 | 1.3 | 0.1 | 51 |
| Gines (1988) | 1 | 0.1 | 52 | 1.1 | 0.1 | 53 |
| Gines (1996) | 0.97 | 0.4 | 97 | 1.02 | 0.55 | 192 |
| Guevara (2012) | 1.3 | 0.2 | 7 | 1.3 | 0.1 | 6 |
| Hamdy (2014) | 1.11 | 0.161 | 25 | 3.02 | 2.58 | 25 |
| Hernandez-Perez (1995) | 0.91 | 0.46 | 8 | 0.94 | 0.39 | 8 |
| Jha (2023) | 0.969 | 0.177 | 25 | 0.99 | 0.182 | 25 |
| Kang (1998) | 1 | 0.5 | 6 | 0.8 | 0.1 | 6 |
| Kar (2023) | 0.96 | 0.53 | 31 | 1.17 | 0.59 | 32 |
| Khan (2015) | 1.3 | 0.6 | 25 | 1.3 | 0.8 | 25 |
| Luca (1995) | 1.1 | 0.4 | 9 | 1.1 | 0.5 | 9 |
| Maiwall (2022) | 1.33 | 0.92 | 50 | 1.55 | 1.26 | 50 |
| Moreau (2002) | 0.803 | 0.068 | 10 | 0.814 | 0.09 | 10 |
| Planas (1990) | 1.1 | 0.1 | 43 | 0.9 | 0.1 | 45 |
| Reddy (2023) | 1.35 | 0.71 | 25 | 1.1 | 0.175 | 25 |
| Salerno (1991) | 1.28 | 0.1 | 27 | 1.08 | 0.1 | 27 |
| Singh (2006) Noradrenaline | 1.06 | 0.3 | 20 | 0.8 | 0.2 | 20 |
| Singh (2006) Terlipressin | 0.93 | 0.2 | 20 | 0.92 | 0.24 | 20 |
| Singh (2008) | 0.98 | 0.25 | 20 | 0.86 | 0.21 | 20 |
| Sola Vera (2003) | 1.01 | 0.366 | 37 | 1.11 | 0.474 | 35 |
| Sola (2018) | 1.2 | 0.3 | 87 | 1.5 | 0.4 | 86 |
| Sort (1999) | 1 | 0.7416 | 55 | 1.4 | 0.6928 | 48 |
| Thevenot (2015) | 0.77 | 0.34 | 91 | 0.96 | 0.75 | 89 |
| Yosry (2019) | 1.23 | 0.16 | 23 | 1.34 | 0.475 | 50 |
| Zaak (2001) | 1.2 | 0.5 | 21 | 1.1 | 0.5 | 14 |

Renal Dysfunction - Serum Sodium:

| Study Name | Serum Sodium (In Albumin Group) | SD | Total Albumin Group Participants | Serum Sodium (In Control Group) | SD | Total Control Group Participants |
| --- | --- | --- | --- | --- | --- | --- |
| Abdul-Khalek (2010) | 134.6 | 1.62 | 68 | 134.7 | 0.95 | 67 |
| Abootalebi (2017) | 134.16 | 3.07 | 36 | 133.58 | 3.05 | 36 |
| Alessandria (2011) | 134 | 4 | 35 | 135 | 5 | 35 |
| AlSebaey (2013) | 126.4 | 3.495 | 50 | 126.9 | 3.57 | 75 |
| Altman (1998) | 134 | 5 | 33 | 133 | 5 | 27 |
| Appenrodt (2008) | 129 | 6.67 | 13 | 129 | 8.15 | 11 |
| Arora (2020) | 131.13 | 6 | 40 | 126.55 | 7.85 | 40 |
| Bari (2012) | 133 | 2.96 | 11 | 134 | 5.19 | 9 |
| Bruno (1992) | 132.01 | 3.74 | 18 | 133 | 4.47 | 17 |
| Chen (2009) | 135.3 | 4.4 | 11 | 103.7 | 5.2 | 9 |
| Fagan (2023) | 138.9 | 2 | 24 | 138.75 | 2.44 | 24 |
| Fassio (1992) | 133 | 5 | 21 | 131 | 8 | 20 |
| Fernandez (2005) | 134 | 7 | 10 | 130 | 8 | 10 |
| Fernandez (2020) | 134.9 | 5.7 | 61 | 133.5 | 5.1 | 57 |
| García-Compean (1993) | 133 | 3.5 | 17 | 133 | 4 | 18 |
| García-Compean (2002) | 136 | 5 | 48 | 135 | 5 | 48 |
| Gentilini (1999) Protocol 1 | 138.05 | 2.62 | 34 | 136.67 | 3.27 | 34 |
| Gines (1987) | 133 | 0.9 | 58 | 130 | 0.8 | 51 |
| Gines (1988) | 133 | 0.7 | 52 | 131 | 1 | 53 |
| Gines (1996) | 132 | 6 | 97 | 131 | 6 | 192 |
| Hamdy (2014) | 131.88 | 3.09 | 25 | 130.2 | 4.1 | 25 |
| Hernandez-Perez (1995) | 134.36 | 5.28 | 8 | 134.94 | 3.95 | 8 |
| Kang (1998) | 132 | 5 | 6 | 135 | 7 | 6 |
| Khan (2015) | 131.5 | 2.4 | 25 | 132.7 | 2.9 | 25 |
| Luca (1995) | 136 | 7 | 9 | 133 | 10 | 9 |
| Moreau (2002) | 132 | 3.16 | 10 | 133 | 3.16 | 10 |
| Planas (1990) | 130 | 6.56 | 43 | 129 | 6.71 | 45 |
| Reddy (2023) | 131.4 | 3.81 | 25 | 128.4 | 2.73 | 25 |
| Salerno (1987) | 132 | 1.1 | 19 | 134 | 1 | 19 |
| Salerno (1991) | 130.4 | 4.16 | 27 | 128.5 | 6.24 | 27 |
| Singh (2006) Noradrenaline | 136.9 | 4.9 | 20 | 135.6 | 5.4 | 20 |
| Singh (2006) Terlipressin | 134.25 | 4.8 | 20 | 132.55 | 4.45 | 20 |
| Singh (2008) | 132.1 | 4.06 | 20 | 133.95 | 4.67 | 20 |
| Sola Vera (2003) | 132.9 | 5.5 | 37 | 130.6 | 6.5 | 35 |
| Sola (2018) | 134 | 3 | 87 | 129 | 5 | 86 |
| Sort (1999) | 134 | 7.28 | 53 | 130 | 7.14 | 51 |
| Yosry (2019) | 129.87 | 3.19 | 23 | 130.88 | 4.22 | 50 |

Circulatory Dysfunction - Plasma Renin Activity:

| Study Name | Plasma Renin (In Albumin Group) | SD | Total Albumin Group Participants | Plasma Renin (In Control Group) | SD | Total Control Group Participants |
| --- | --- | --- | --- | --- | --- | --- |
| Abdul-Khalek (2010) | 13.91 | 1.38 | 68 | 14.54 | 1.12 | 67 |
| Alessandria (2011) | 9 | 6.1 | 35 | 9 | 9 | 35 |
| Arora (2020) | 25.8 | 12.81 | 40 | 35.7 | 11.78 | 40 |
| Baik (2000) | 14.21 | 15.63 | 11 | 18.57 | 15.74 | 12 |
| Bari (2012) | 23.6 | 12.1 | 11 | 14.9 | 11.9 | 8 |
| Fassio (1992) | 7.9 | 7 | 12 | 8.7 | 4 | 12 |
| Fernandez (2005) | 3.1 | 3.4 | 10 | 16.8 | 24.6 | 10 |
| García-Compean (2002) | 13.3 | 12.6 | 16 | 17 | 10 | 19 |
| Gines (1988) | 6.5 | 6.86 | 24 | 10.8 | 7.84 | 24 |
| Gines (1996) | 9.3 | 10.9 | 97 | 12.15 | 13.1 | 192 |
| Guevara (2012) | 1.3 | 1.7 | 46 | 3.1 | 7.1 | 51 |
| Hamdy (2014) | 4.11 | 0.74 | 25 | 4.2 | 0.76 | 25 |
| Hernandez-Perez (1995) | 2.14 | 1.82 | 8 | 3.03 | 2.72 | 8 |
| Lata (2007) | 4.3 | 1.1 | 25 | 1.1 | 1.6 | 24 |
| Luca (1995) | 11.3 | 10.3 | 9 | 11.4 | 11 | 9 |
| Planas (1990) | 10 | 1.5 | 6 | 13.5 | 2.5 | 18 |
| Reddy (2023) | 22.86 | 14.63 | 25 | 25.38 | 12.89 | 25 |
| Salerno (1987) | 9.5 | 3.8 | 12 | 13.5 | 1.3 | 13 |
| Salerno (1991) | 14.3 | 10.91 | 27 | 12.4 | 7.27 | 27 |
| Salman (2016) | 7.23 | 6.21 | 100 | 9.61 | 6.01 | 100 |
| Singh (2006) Noradrenaline | 22.02 | 10.15 | 20 | 20.78 | 9.41 | 20 |
| Singh (2006) Terlipressin | 20.33 | 12.87 | 20 | 20.11 | 10 | 20 |
| Singh (2008) | 45.90 | 8.59 | 20 | 41.39 | 10.21 | 20 |
| Sola Vera (2003) | 5 | 5.2 | 37 | 8.3 | 7.9 | 35 |
| Sort (1999) | 4 | 4.53 | 57 | 9.4 | 7.85 | 51 |
| Zaak (2001) | 17.7 | 32.93 | 14 | 16.75 | 31.73 | 13 |

**5. REPORTING BIAS (FUNNEL PLOT):**

**Funnel Plot Showing Reporting Bias for Mortality as an Outcome**

Bias Indicators:

Begg-Mazumdar: Kendall's -0.054422  P = 0.5772

Egger: bias = -0.667761 (95% CI = -1.263882 to -0.071639)  P = 0.0289

Harbord-Egger: bias = -0.616212 (92.5% CI = -1.21179 to -0.020634)  P = 0.0658

Statistical Heterogenicity:

Cochran Q = 61.351103  (df = 48)  P = 0.0934

I_2_ (inconsistency) = 21.8% (95% CI = 0% to 45.1%)

Chi^2^ (test odds ratio differs from 1) = 9.644756  (df = 1)  P = 0.0019

**6. META-ANALYSIS OF BINARY OUTCOMES:**

**A. Clinical Indication for HAS use in patients with hepatic encephalopathy and liver cirrhosis**

Favours Alternatives

Favours Albumin

Chi^2^ (test odds ratio differs from 1) = 2.590007  (df = 1)  P = 0.1075

Cochran Q = 34.322088  (df = 32)  P = 0.357

**B. Clinical Indication for HAS use in patients with GI bleeding and liver cirrhosis**

Favours Albumin

Favours Alternatives

Chi^2^ (test odds ratio differs from 1) = 0.101756  (df = 1)  P = 0.7497

Cochran Q = 19.853698  (df = 26)  P = 0.7984

**C. Clinical Indication for HAS use in patients with portal hypertensive bleeding and liver cirrhosis**

Favours Albumin

Favours Alternatives

Chi^2^ (test odds ratio differs from 1) = 0.241857 (df = 1) P = 0.6229

Cochran Q = 8.707834  (df = 10)  P = 0.56

**D. Clinical Indication for HAS use in patients with SBP and liver cirrhosis**

Favours Albumin

Favours Alternatives

Chi^2^ (test odds ratio differs from 1) = 1.494783  (df = 1)  P = 0.2215

Cochran Q = 11.90579  (df = 18)  P = 0.8521

**E. Clinical Indication for HAS use in patients with Non-SBP and liver cirrhosis**

Favours Alternatives

Favours Albumin

Chi^2^ (test odds ratio differs from 1) = 0.559102  (df = 1)  P = 0.4546

Cochran Q = 34.272045  (df = 24)  P = 0.0799

**F. Clinical Indication for HAS use in patients with the need for paracentesis and liver cirrhosis**

Favours Alternatives

Favours Albumin

Chi^2^ (test odds ratio differs from 1) = 3.389473  (df = 1)  P = 0.0656

Cochran Q = 6.824858  (df = 5)  P = 0.234

**G. Forest Plot showing the effect of HAS treatment on multi-organ failure and liver cirrhosis**

Favours Alternatives

Favours Albumin

Chi^2^ (test odds ratio differs from 1) = 0.880496 (df = 1) P = 0.3481

Cochran Q = 3.42777  (df = 4)  P = 0.4889

**H. Forest Plot showing the effect of HAS treatment on Acute-On-Chronic liver failure and liver cirrhosis**

Favours Albumin

Favours Alternatives

Chi^2^ (test odds ratio differs from 1) = 1.934414 (df = 1) P = 0.1643

Cochran Q = 0.438768  (df = 2)  P = 0.803

**7. META-ANALYSIS OF CONTINIOUS OUTCOMES:**

**A. EFFECT OF HAS TREATMENT ON SERUM CREATININE**

Favours Alternatives

Favours Albumin

Z (test wmd+ differs from 0) = -1.232169  P = 0.2179

Cohran Q = 411.191348  (df = 39)  P < 0.0001

**B. EFFECT OF HAS TREATMENT ON SERUM SODIUM**

Favours Alternatives

Favours Albumin

Z (test wmd+ differs from 0) = 3.483993  P = 0.0005

Cohran Q = 540.648516  (df = 36)  P < 0.0001

**C. EFFECT OF HAS TREATMENT ON PLASMA RENIN ACTIVITY**

Favours Alternatives

Favours Albumin

Z (test wmd+ differs from 0) = -2.959801  P = 0.0031

Cohran Q = 164.571592  (df = 25)  P < 0.0001

**8. REFERENCES OF ALL RANDOMISED CONTROLLED TRIALS ^1-67^**

1. Abdel-Khalek EE, Arif SE. Randomized trial comparing human albumin and hydroxyethyl starch 6% as plasma expanders for treatment of patients with liver cirrhosis and tense ascites following large volume paracentesis. Arab Journal of Gastroenterology. 2010;11(1):24-29. doi:10.1016/j.ajg.2010.01.006
2. Abootalebi A, Khazaei S, Minakari M, Nasr-Isfahani M, Esmailian M, Heydari F. Comparing the Effects of Hydroxyethyl Starch and Albumin in Cirrhotic Patients with Tense Ascites; a Randomized Clinical Trial. Adv J Emerg Med. Fall 2017;1(1):e7. doi:10.22114/AJEM.v1i1.17
3. Alessandria C, Elia C, Mezzabotta L, et al. Prevention of paracentesis-induced circulatory dysfunction in cirrhosis: standard vs half albumin doses. A prospective, randomized, unblinded pilot study. Dig Liver Dis. Nov 2011;43(11):881-6. doi:10.1016/j.dld.2011.06.001
4. Alsebaey A, Abdel-Razek W, Bassuni A, Rewisha E, Khalil M, Waked I. Prevention of paracentesis-induced circulatory dysfunction. Egyptian Liver Journal. 2013;3(4):118-125. doi:10.1097/01.Elx.0000433597.15423.45
5. Claude Altman BB, Dominique Roulote, René-Louis Vitted and Olivier Inke. Randomized comparative multicenter study of hydroxyethyl starch versus albumin as a plasma expander in cirrhotic patients with tense ascites treated with paracentesis. European Journal of Gastroenterology. 1998,; Vol 10 (No 1):5-10.
6. Appenrodt B, Wolf A, Grunhage F, et al. Prevention of paracentesis-induced circulatory dysfunction: midodrine vs albumin. A randomized pilot study. Liver Int. Aug 2008;28(7):1019-25. doi:10.1111/j.1478-3231.2008.01734.x
7. Arora V, Vijayaraghavan R, Maiwall R, et al. Paracentesis-Induced Circulatory Dysfunction With Modest-Volume Paracentesis Is Partly Ameliorated by Albumin Infusion in Acute-on-Chronic Liver Failure. Hepatology. Sep 2020;72(3):1043-1055. doi:10.1002/hep.31071
8. Bari K, Minano C, Shea M, et al. The combination of octreotide and midodrine is not superior to albumin in preventing recurrence of ascites after large-volume paracentesis. Clin Gastroenterol Hepatol. Oct 2012;10(10):1169-75. doi:10.1016/j.cgh.2012.06.027
9. Bertrán XF-B, F.; Planas, R.; Cabré, E.; Morillas, R.; De León, R.; Guilera, M.; Quer, J. C.;, Llovet JMD, E., y Gassull, M. A. Repercusión sobre el estado nutricional energético-proteico de la paracentesis total asociada a la infusión de albúmina o dextrano-70 en el tratamiento de la ascitis a tensión en la cirrosis hepática. Rev Esp Enf Digest. 1991;79(5 ):320-324.
10. S Bruno M, MRomagnoni, PMBattezzati, S Rossi, AChiesa, MPodda. Comparison of spontaneous ascites filtration and reinfusion with total paracentesis with intravenous albumin infusion in cirrhotic patients with tense ascites. BMJ. 1992;304:1655-8.
11. Caraceni P, Riggio O, Angeli P, et al. Long-term albumin administration in decompensated cirrhosis (ANSWER): an open-label randomised trial. Lancet. Jun 16 2018;391(10138):2417-2429. doi:10.1016/S0140-6736(18)30840-7
12. Chen TA, Tsao YC, Chen A, et al. Effect of intravenous albumin on endotoxin removal, cytokines, and nitric oxide production in patients with cirrhosis and spontaneous bacterial peritonitis. Scand J Gastroenterol. 2009;44(5):619-25. doi:10.1080/00365520902719273
13. China L, Freemantle N, Forrest E, et al. A Randomized Trial of Albumin Infusions in Hospitalized Patients with Cirrhosis. N Engl J Med. Mar 4 2021;384(9):808-817. doi:10.1056/NEJMoa2022166
14. Vesna Degoricija VZ-R, Jasminka Marout, Siniša Šefer, Branko Troskot. Clinical and Neurohumoral Response to Posture, Physical Exercise, and Ascites Treatment in Child-Pugh C Liver Cirrhosis: Randomized Prospective Trial. Croatian Medical Journal. 2003;44:178-186.
15. Devisetty JV, Mallick B, Praharaj D, et al. A Study of Impact of Fixed-Dose Albumin Infusion on Outcome in Patients With Cirrhosis and Infection: A Randomized Open-label Clinical Trial. J Clin Exp Hepatol. Jan-Feb 2024;14(1):101270. doi:10.1016/j.jceh.2023.08.006
16. Fagan A, Gavis EA, Gallagher ML, et al. A double-blind randomized placebo-controlled trial of albumin in outpatients with hepatic encephalopathy: HEAL study. J Hepatol. Feb 2023;78(2):312-321. doi:10.1016/j.jhep.2022.09.009
17. Eduardo Fassio’ RT, Graciela Landeira’, Raquel Abecasis2, Marta Salemnel, Ana Podesta2, Patricia Rodriguez’, Diana Levi2 and David Kravetz’. Paracentesis with Dextran 70 vs. paracentesis wit albumin in cirrhosis with tense ascites. Results of a randomized study. Journal of Hepatology,. 1992;14:310-316.
18. Fernandez J, Monteagudo J, Bargallo X, et al. A randomized unblinded pilot study comparing albumin versus hydroxyethyl starch in spontaneous bacterial peritonitis. Hepatology. Sep 2005;42(3):627-34. doi:10.1002/hep.20829
19. Fernandez J, Angeli P, Trebicka J, et al. Efficacy of Albumin Treatment for Patients with Cirrhosis and Infections Unrelated to Spontaneous Bacterial Peritonitis. Clin Gastroenterol Hepatol. Apr 2020;18(4):963-973 e14. doi:10.1016/j.cgh.2019.07.055
20. Garcia-Compean D, Zacarias Villarreal J, Bahena Cuevas H, et al. Total therapeutic paracentesis (TTP) with and without intravenous albumin in the treatment of cirrhotic tense ascites: a randomized controlled trial. Liver. Oct 1993;13(5):233-8. doi:10.1111/j.1600-0676.1993.tb00637.x
21. García-Compean D, Blanc P, Larrey D, et al. Treatment of cirrhotic tense ascites with Dextran-40 versus albumin associated with large volume paracentesis: a randomized controlled trial. Annals of Hepatology. 2002;1(1):29-35. doi:10.1016/s1665-2681(19)32189-1
22. Paolo Gentilini VC-R, Giuseppe Di Fiore, Roberto Giulio Romane, Giampiero Buzzelli, Massimo Pinzani, Giorgio La Villa and Giacbmo Laffi. Albumin improves the response to diuretics in patients with cirrhosis and ascites: results of a randomized, controlled trial. Journal of Hepatology. 1999; 30:639-645.
23. PERE GINES VA, ENRIQUE QUINTERO,, RAMON PLANAS FB, JUAN CABRERA,, ANTON1 RIMOLA JV, JORDI CAMPS,, WLADIMIRO JIMENEZ RM, JOAN GAYA, JOAN RODES. Comparison of Paracentesis and Diuretics in the Treatment of Cirrhotics With Tense Ascites Results of a Randomized Study. GASTROENTEROLOGY 1987;93:234-41.
24. Gines P, Tito L, Arroyo V, et al. Randomized comparative study of therapeutic paracentesis with and without intravenous albumin in cirrhosis. Gastroenterology. Jun 1988;94(6):1493-502. doi:10.1016/0016-5085(88)90691-9
25. ANGELS GINES GFE, ALBERTO MONESCILLO, CARME VILA, EUGENI DOMENECH, RAQUEL ABECASIS, PAOLO ANGELI, LUIS RUIZ–DEL–ARBOL, RAMON PLANAS, RICARD SOLA, PERE GINES, RUBEN TERG, LUIS INGLADA,PERE VAQUE, FRANCESCO SALERNO,VICTOR VARGAS,GERARDO CLEMENTE, JOAN C. QUER, WLADIMIRO JIME, NEZ VA, JUAN RODE. Randomized Trial Comparing Albumin, Dextran 70, and Polygeline in Cirrhotic Patients With Ascites Treated by Paracentesis. GASTROENTEROLOGY 1996;111 1002–1010.
26. Guevara M, Terra C, Nazar A, et al. Albumin for bacterial infections other than spontaneous bacterial peritonitis in cirrhosis. A randomized, controlled study. J Hepatol. Oct 2012;57(4):759-65. doi:10.1016/j.jhep.2012.06.013
27. Hassan Hamdy AAE, Ahmed Hassan, Omayma Hassanin. Comparison of Midodrine and Albumin in the Prevention of Paracentesis-induced Circulatory Dysfunction in Cirrhotic Patients. A Randomized Pilot Study. Journal of Clinical Gastroenterolgy. 2014;48(2 ):184-188.
28. ROLANDO ERNESTO HERNANDEZ PEREZ JRAR, JOSE MARIANO HERNANDEZ LOPEZ, TOMAS GOMEZ MAGANDA SILVA. Paracentesis Masiva con Reposicion de Dextran 70 vs Albumina en Pacientes Cirroticos con Ascitis a Tension. Rev Gastroentrology Mex. 1995;60(1):22-26.
29. Jalan R, Mookerjee R, Cheshire L, Williams R, Davies N. [232] Albumin Infusion for Severe Hyponatremia in Patients with Refractory Ascites: A Randomized Clinical Trial. Journal of Hepatology. 2007;46doi:10.1016/s0168-8278(07)61830-3
30. U. C. Jha SKM, Prabhat Kumar. COMPARATIVE STUDY OF THE EFFECT OF LARGE VOLUME PARACENTESIS ON CEREBRAL AND RENAL FUNCTION IN PATIENTS WITH CIRRHOSIS OF THE LIVER WITH TENSE ASCITES AFTER EITHER ALBUMIN OR POLYGELINE INFUSION. International Journal of Academic Medicine and Pharmacy. 2023;5(3):1499-1509. doi:10.47009/jamp.2023.5.3.305
31. Kyung Eui Kang BHK, Seok Ho Dong, Hyo Jong Kim, Young Woon Chang, Joung Il Lee, Rin Chang. The effeciacy of hydroxyethyl starch as plasma expander in cirrhotic patients with tense ascites treated with large volume paracentesis The Korean Journal of gastroentrology. 1998;31(5):651-660.
32. Kar PS, Venishetty S, Laroia ST, et al. Tolerance of standard dose albumin infused over 6 hrs for treatment of spontaneous bacterial peritonitis-A randomized controlled trial. Indian J Gastroenterol. Aug 2023;42(4):505-516. doi:10.1007/s12664-023-01389-x
33. MUTI ULLAH KHAN IUR, MUHAMMAD LATIF. Hemaccel as a Cheaper Alternative to Human Albumin for Plasma Expansion during Paracentesis in Cirrhotic patients. The Pakistan Journal of Medical & Health Sciences 2015;9(3):948-951.
34. Jan Lata ZkM, Tomas Fejfar, Petr Zdenēk, Radan Bruha, Vaclav Safka, Petr Hülek, Vaclav Hejda, Jiri Dolina, Jiri Stehlik, Igor Tozzie. The efficacy of terlipressin in comparison with albumin in the prevention of circulatory changes after the paracentesis of tense ascites--a randomized multicentric study. Hepato-gastroenterology. 2007;54(79):1930-1933.
35. ANGELO LUCA JCG-P, JAUME BOSCH, FAUST FEU, WLADIMIRO JIMIENEZ, ANGELS GINES, MERCEDES FERNANDEZ, ANGELS ESCORSELL, VICENTE ARROYO, JOAN RODES. Beneficial on the Effects of Intravenous Albumin Infusion and Humeral Changes After Total Paracentesis. Hepatology. 1995;22(3):753-758.
36. Maiwall R, Kumar A, Pasupuleti SSR, et al. A randomized-controlled trial comparing 20% albumin to plasmalyte in patients with cirrhosis and sepsis-induced hypotension [ALPS trial]. J Hepatol. Sep 2022;77(3):670-682. doi:10.1016/j.jhep.2022.03.043
37. Rakhi Maiwall NC, Ashinikumar Kumar Hidam, Sherin Thomas, Anupam Kumar, Jaswinder, Maras NS, Jaya Benjamin, Sukriti Sukriti, Shiv Kumar Sarin. E F F I C A C Y O F T A R G E T E D A N D R E S P O N S E - G U I D E D A L B U M I N T H E R A P Y V E R S U S S T A N D A R D M E D I C A L T R E A T M E N T I N O U T C O M E S O F R E C U R R E N T A S C I T E S I N P A T I E N T S W I T H D E C O M P E N S A T E D C I R R H O S I S. American Association for the Study of Liver Diseases - Hepatology. 2023;(S1336):abstract 3014-A.
38. Rakhi Maiwall SSRP, Sherin Thomas, Jaswinder singh Maras, Sukriti Baweja,, Anupam Kumar JB, Harsh Vardhan Tevethia, Kanika Jain, Puja Bhatia Kapoor, Bindiya Arora, Varsha Shasthry, Shiv Kumar Sarin. TARGETING LOWER SERUM ALBUMIN WITH LONG TERM ALBUMIN ADMINISTRATION IMPROVES OUTCOMES OF RECURRENT ASCITES IN PATIENTS WITH DECOMPENSATED CIRRHOSIS- FINAL RESULTS OF THE RANDOMISED CONTROLLED TRIAL. American Association for the Study of Liver Diseases - Hepatology. 2024;(S542):abstract 729.
39. R Moreau TA, B Condat, C de Kerguenec, F Pessione, B Bernard, T Poynard,, M Binn JG, D Valla, D Lebrec. Comparison of the effect of terlipressin and albumin on arterial blood volume in patients with cirrhosis and tense ascites treated by paracentesis: a randomised pilot study. BMJ - Gut - Liver and biliary disease. 2002;50(1):90–94.
40. Moreau R, Valla DC, Durand-Zaleski I, et al. Comparison of outcome in patients with cirrhosis and ascites following treatment with albumin or a synthetic colloid: a randomised controlled pilot trail. Liver Int. Feb 2006;26(1):46-54. doi:10.1111/j.1478-3231.2005.01188.x
41. Naidu M, Mishra S, Nath P. Sa1590 COMPARISON OF LOW DOSE ALBUMIN WITH STANDARD DOSE ALBUMIN FOR PREVENTION OF PARACENTESIS INDUCED CIRCULATORY DYSFUNCTION AFTER LARGE VOLUME PARACENTESIS IN PATIETNS WITH DECOMPENSATED CIRRHOSIS OF LIVER. Gastroenterology. 2024;166(5)doi:10.1016/s0016-5085(24)04106-4
42. O’Leary JG, Perricone G, Laleman W, et al. LBO-003 Efficacy and safety of long-term human albumin therapy in cirrhotic patients with acute decompensation and ascites: topline results of the PRECIOSA trial. Journal of Hepatology. 2025;82doi:10.1016/s0168-8278(25)00312-5
43. Philips CA, Maiwall R, Sharma MK, et al. Comparison of 5% human albumin and normal saline for fluid resuscitation in sepsis induced hypotension among patients with cirrhosis (FRISC study): a randomized controlled trial. Hepatol Int. Aug 2021;15(4):983-994. doi:10.1007/s12072-021-10164-z
44. Planas R, Gines P, Arroyo V, et al. Dextran-70 versus albumin as plasma expanders in cirrhotic patients with tense ascites treated with total paracentesis. Results of a randomized study. Gastroenterology. Dec 1990;99(6):1736-44. doi:10.1016/0016-5085(90)90481-f
45. Sujith Reddy JSN, Jagtap N, Kalpala R, et al. Midodrine versus Albumin to Prevent Paracentesis Induced Circulatory Dysfunction in Acute on Chronic Liver Failure Patients in the Outpatient Clinic-a Randomized Controlled Trial. J Clin Exp Hepatol. Jul-Aug 2023;13(4):576-585. doi:10.1016/j.jceh.2023.01.009
46. Roberto Giulio Romanelli GLV, Giuseppe Barletta, Francesco Vizzutti, Fabio Lanini, Umberto Arena, Vieri Boddi, Roberto Tarquini, Pietro Pantaleo, Paolo Gentilini, Giacomo Laffi. Long-term albumin infusion improves survival in patients with cirrhosis and ascites: An unblinded randomized trial. World Journal of Gastroenterology. 2006;12(9):1403-1407.
47. Francesco Salerno SB, Pierluigi Incerti, Silvana Tempini, Bruno Restelli, Savino Bruno, Giorgio Bellati, Luigi Roffi. Repeated paracentesis and i.v. albumin infusion to treat 'tense' ascites in cirrhotic patients. A safe alternative therapy. Journal of Hepatology. 1987;5:102-108.
48. Salerno F, Badalamenti S, Lorenzano E, Moser P, Incerti P. Randomized comparative study of hemaccel vs. albumin infusion after total paracentesis in cirrhotic patients with refractory ascites. Hepatology. 1991;13(4):707-713. doi:10.1002/hep.1840130416
49. Salman TA, Edrees AM, El-Said HH, El-Abd OL, El-Azab GI. Effect of different therapeutic modalities on systemic, renal, and hepatic hemodynamics and short-term outcomes in cirrhotic patients with spontaneous bacterial peritonitis. Eur J Gastroenterol Hepatol. Jul 2016;28(7):777-85. doi:10.1097/MEG.0000000000000635
50. Sharma BC, Singh J, Srivastava S, et al. Randomized controlled trial comparing lactulose plus albumin versus lactulose alone for treatment of hepatic encephalopathy. J Gastroenterol Hepatol. Jun 2017;32(6):1234-1239. doi:10.1111/jgh.13666
51. Simon-Talero M, Garcia-Martinez R, Torrens M, et al. Effects of intravenous albumin in patients with cirrhosis and episodic hepatic encephalopathy: a randomized double-blind study. J Hepatol. Dec 2013;59(6):1184-92. doi:10.1016/j.jhep.2013.07.020
52. Singh V, Kumar B, Nain CK, et al. Noradrenaline and albumin in paracentesis-induced circulatory dysfunction in cirrhosis: a randomized pilot study. J Intern Med. Jul 2006;260(1):62-8. doi:10.1111/j.1365-2796.2006.01654.x
53. Singh V, Kumar R, Nain CK, Singh B, Sharma AK. Terlipressin versus albumin in paracentesis-induced circulatory dysfunction in cirrhosis: a randomized study. J Gastroenterol Hepatol. Jan 2006;21(1 Pt 2):303-7. doi:10.1111/j.1440-1746.2006.04182.x
54. Singh V, Dheerendra PC, Singh B, et al. Midodrine versus albumin in the prevention of paracentesis-induced circulatory dysfunction in cirrhotics: a randomized pilot study. Am J Gastroenterol. Jun 2008;103(6):1399-405. doi:10.1111/j.1572-0241.2008.01787.x
55. Sola E, Sole C, Simon-Talero M, et al. Midodrine and albumin for prevention of complications in patients with cirrhosis awaiting liver transplantation. A randomized placebo-controlled trial. J Hepatol. Dec 2018;69(6):1250-1259. doi:10.1016/j.jhep.2018.08.006
56. Sola-Vera J, Minana J, Ricart E, et al. Randomized trial comparing albumin and saline in the prevention of paracentesis-induced circulatory dysfunction in cirrhotic patients with ascites. Hepatology. May 2003;37(5):1147-53. doi:10.1053/jhep.2003.50169
57. PAU SORT MINAVIAR, X AVIER A LDEGUER, R AMON PLANAS, LUIS RUIZ- DE-A RBOL, L LUIS C ASTELLS, V ICTOR V ARGAS, GERMÁN SORIANO, MÓNICA GUEVARA, PERE GINÈS, JOAN RODÉS. EFFECT OF INTRAVENOUS ALBUMIN ON RENAL IMPAIRMENT AND MORTALITY IN PATIENTS WITH CIRRHOSIS AND SPONTANEOUS BACTERIAL PERITONITIS. The New England Journal of Medicine. 1999;341(6):403-409.
58. Surabhi. Effect of long-term albumin infusion in patient with cirrhosis and ascites. Journal of Clinical and Experimental Hepatology. 2024;14doi:10.1016/j.jceh.2024.102066
59. Kazuo TARAO KIA. Influence of long-term administration of serum albumin on the prognosis of liver cirrhosis in man. Tokai journal of experimental and clinical medicine. 1983;8(1):71-8.
60. Thevenot T, Bureau C, Oberti F, et al. Effect of albumin in cirrhotic patients with infection other than spontaneous bacterial peritonitis. A randomized trial. J Hepatol. Apr 2015;62(4):822-30. doi:10.1016/j.jhep.2014.11.017
61. Ventura-Cots M, Simon-Talero M, Poca M, et al. Effects of Albumin on Survival after a Hepatic Encephalopathy Episode: Randomized Double-Blind Trial and Meta-Analysis. J Clin Med. Oct 23 2021;10(21)doi:10.3390/jcm10214885
62. PATRICIA WILKINSON SS. THE EFFECT OF REPEATED ALBUMIN INFUSIONS IN PATIENTS WITH CIRRHOSIS. The Lancet. 1962:125-129.
63. Xue HP, Lin B, Mo JZ, Li JQ. Effect of albumin infusion on preventing the deterioration of renal function in patients with spontaneous bacterial peritonitis. Chinese Journal of Digestive Diseases. 2002;3(1):32-34. doi:10.1046/j.1443-9573.2002.00062.x
64. Yosry A, Soliman ZA, Eletreby R, Hamza I, Ismail A, Elkady MA. Oral midodrine is comparable to albumin infusion in cirrhotic patients with refractory ascites undergoing large-volume paracentesis: results of a pilot study. Eur J Gastroenterol Hepatol. Mar 2019;31(3):345-351. doi:10.1097/MEG.0000000000001277
65. Zaak D PK, Kuhn R..l. Prospective study comparing human albumin vs. reinfusion of ultrafiltrate-ascitic fluid after total para- centesis in cirrhotic patients with tense ascites. Zeitschrift für Gastroenterologie. 2001;39(1):5-10.
66. Zhao Jiang YC, Wang Dajun, LI Xingwu. Mannitolum infusion on cirrhotic patients with tense ascites treated by paracentesis. The chinese medical journal. 2000;113(1)
67. S. K. Baik HSK, I. H. Kim and S. O. Kwon. Hemodynamic changes after single large volume paracentesis (SLVP) in cirrhosis with tense ascites: focusing on the effect of albumin as a plasma expander. The Korean journal of medicine. 2000;58(3):276-282.
